# Argument-based human–AI collaboration for supporting behavior change to improve health

**DOI:** 10.3389/frai.2023.1069455

**Published:** 2023-02-16

**Authors:** Kaan Kilic, Saskia Weck, Timotheus Kampik, Helena Lindgren

**Affiliations:** Department of Computing Science, Umeå University, Umeå, Sweden

**Keywords:** formal argumentation dialogues, behavior change, digital companion, value-based argumentation, argumentation schemes, user-modeling, Human-Centered Artificial Intelligence, health promotion

## Abstract

This article presents an empirical requirement elicitation study for an argumentation-based digital companion for supporting behavior change, whose ultimate goal is the promotion and facilitation of healthy behavior. The study was conducted with non-expert users as well as with health experts and was in part supported by the development of prototypes. It focuses on human-centric aspects, in particular user motivations, as well as on expectations and perceptions regarding the role and interaction behavior of a digital companion. Based on the results of the study, a framework for person tailoring the agent's roles and behaviors, and argumentation schemes are proposed. The results indicate that the extent to which a digital companion argumentatively challenges or supports a user's attitudes and chosen behavior and how assertive and provocative the companion is may have a substantial and individualized effect on user acceptance, as well as on the effects of interacting with the digital companion. More broadly, the results shed some initial light on the perception of users and domain experts of “soft,” meta-level aspects of argumentative dialogue, indicating potential for future research.

## 1. Introduction

Artificially intelligent agents in the form of digital assistants, or companions (Torous et al., 2018), are to an increasing extent being developed for supporting individuals with improving health by changing unhealthy behavior. However, each individual has different motives for attempting a change of behavior and different reasons for why they do not achieve the desired behavior. These motives and reasons can be formulated as arguments, which can potentially be used as the basis for argument-based dialogues between an individual and a digital companion. Moreover, users may have different perceptions of how an agent could collaborate and provide support in the process, which may affect how argument-based dialogues with a digital companion can unfold.

Although there are plenty of examples of behavior change support applications, few apply computational argumentation frameworks as the foundation for organizing motives in favor and against what to do to promote health and in the reasoning in deliberative dialogues between the human and a digital agent.

The purpose of the research presented in this article is to explore from a user's perspective and from the perspectives of experts on behavior change a digital companion with which the user can have argument-based dialogues in the process of behavior change, which the user can tailor to adhere to their expectations regarding roles and types of support in the dialogues. The aim is to provide the user with means to collaborate with the digital agent to ultimately become empowered and supported in their pursue of their goals to improve their health. Research presented, in this article, is consequently an example of Human-Centered Artificial Intelligence (HCAI), which is defined by Nowak et al. (2018) as AI that collaborates with a human, “enhancing their capabilities, and empowering them to better achieve their goals.”

Our study explores the following research questions:

- What are people's expectations of a digital coach or companion in terms of roles and behaviors, and argument-based support?
- How can the agent's roles and behavior, and the argument-based dialogue promoting health be tailored to individuals' expectations and level of readiness for the change?

The main contributions to the field of HCAI are (i) increased knowledge about how people view argument-based support through digital companions for promoting healthy lifestyles, (ii) an argumentation-based framework for tailoring a digital agent's roles and behaviors, and (iii) a novel application of argumentation schemes for tailoring a digital companion's role and behavior and for switching between or merging roles. The article exemplifies how computational argumentation provides the foundation for HCAI for supporting behavior change to improve health.

The remainder of the article is organized as follows. First, the conducted research is contextualized and an overview of related work in computational argumentation and human–computer interaction is provided in section 2. Next, the methodology applied in the studies conducted is presented in section 3. The results are provided in section 4 and are discussed in section 5. Conclusion is provided in section 6.

## 2. Background and related work

The research presented in this article is conducted as a part of a research project exploring digital companions as social actors related to managing stress, and the research program STAR-C, which aims to develop a digital coach for promoting healthy lifestyle habits targeting physical activity, nutrition, alcohol consumption, tobacco use, and stress (Lindgren et al., 2020; Ng et al., 2021). The STAR-C program builds on and extends the Västerbotten Health Intervention Program (VIP) in which the population in the healthcare regions are invited to a health checkup that includes motivational interviewing with a specially trained nurses when turning 40, 50, and 60 years old (Hörnsten et al., 2014). The VIP is successful in reducing premature cardiovascular disease mortality and extending a healthy life in a cost-effective manner and has become a health promotion model also for other regions (Blomstedt et al., 2015; Lindholm et al., 2018).

The concept of digital companions for maintaining a healthy lifestyle and goal achievement is increasingly gaining attention; it is, for example, applied and studied in the context of professional work support, education, stress management, healthcare, and behavior change (Jang and Kim, 2020; Braun et al., 2021; Spirig et al., 2021; Weber et al., 2021; Manning et al., 2022). All the facets, however, converge on similar topics, such as assessing the user's context or learning more about the user's habits in the interest of providing personalized support to address a specific problem. Such knowledge about the user is then embedded in a user model, which guides the system in tailoring its behavior to an individual's needs and preferences (Kobsa, 1990). Increasingly, the importance of building an artificial theory of mind (ToM) in digital and robotic companions similar to what humans do about others in order to understand and predict others' behaviors and intentions, has been pointed out, recently as being one of three grand challenges for human–AI interaction (Yang et al., 2018) that is instrumental to human-centered AI (Nowak et al., 2018). To achieve this, models are required that integrate different aspects such as episodic memory, empathy, hierarchical models of activity, and tasks to advance the capabilities (Steels, 2020).

The person-tailored argumentation-based decision-support system developed as a part of this research rests on complementary theoretical frameworks developed within different fields of research to encompass the human-centric approach: (i) on human activity (Kaptelinin and Nardi, 2006; Lindgren and Weck, 2022), (ii) motivation and behavior change (Ryan and Deci, 2000; Prochaska et al., 2015; Lindgren and Weck, 2021), (iii) argumentation theory (Walton and Krabbe, 1995; Bench-Capon, 2002; Walton et al., 2008), and (iv) formal argument-based dialogues for reasoning about health (Atkinson et al., 2006; Lindgren et al., 2020; Guerrero and Lindgren, 2021a,b).

Goal setting is one of the most important personalization feature for promoting behavior change (op den Akker et al., 2014). Using goal setting along with feedback for motivational effectiveness is a very simple yet potent approach to induce a sense of accomplishment and behavior change in users (Locke and Latham, 1984; Lunenburg, 2011). It also leads to a better performance in the attempts to complete the goals and gain motivation (Latham and Locke, 1991). According to Locke and Latham (1984), introducing challenging but specific and achievable goals lead to clearer expectations of what a person must do for behavior change. According to Ryan and Deci (2000), motivation is “to be moved to do something” and a need for *autonomy, competence* and *relatedness* are the attributes that need to be satisfied in order to bring about intrinsic motivation in a person or, possibly, cause an orientation shift in those who were initially not intrinsically motivated. Internalization and the accommodation of the three attributes of motivation are important for user acceptance, sustainable behavior change, and obtaining goal commitment, which are heavily related to contextual and informed feedback communicated to the individual (Locke and Latham, 1984; Ryan and Deci, 2000; Jang and Kim, 2020).

Activity theory guides in this study the organization of arguments based on their content, in addition to providing the framework for understanding the human in interaction with AI systems. Activity theory defines purposeful human activity as being directed by a *motive*, responding to a human's underlying *needs* (Kaptelinin and Nardi, 2006), and composed of an hierarchy of goal-directed *actions*. At the lowest level, the *operational* tasks are found, those that are internalized and conducted without cognitive effort. Large part of a human's habits are governed at this level, without consciously deliberating on why or how to do a particular task (walking, taking the elevator instead of the stairs, sitting down, taking the car to work, etc.). In setting goals and deliberating on what to do to promote healthy habits, e.g., in motivational dialogues with a nurse or in argument-based dialogues with a digital companion, moving between the levels of the activity hierarchy is necessary to find the grounds for why doing a particular action or activity, to formulate the motivating arguments relevant and importance for the individual. The connection between needs, long-term goals, and short-term goals was explored by Lindgren and Weck (2022), and a model of activity encompassing the building blocks for arguments across the levels of activity was defined. Furthermore, to identify the factors affecting an individual's motivation to change behavior, a model of the behavior change progress was built based on the most influential theories on motivation and behavior change (Lindgren and Weck, 2021). These two models build the basis for a user model, or ToM, for the digital companion to use in dialogues with the individual in this study.

Argumentation theory and its application in machine reasoning is an established research field encompassing formal frameworks for constructing, analyzing, and evaluating arguments, typically organized in argumentative dialogues for different purposes, e.g., for generating new knowledge, deliberating on what to do, or to persuade another agent (Walton and Krabbe, 1995). A notable foundational work on computational argumentation is Dung's study on abstract argumentation, in which arguments and conflicts between them are modeled as directed graphs—so-called argumentation frameworks (Dung, 1995).

In order to embed various factors affecting natural dialogues, formal frameworks have been developed which handle values (Bench-Capon, 2002), preferences (Amgoud and Cayrol, 2013), and audiences (Bench-Capon et al., 2007). Bench-Capon (2002) introduced *value-based* argumentation frameworks by adding a set of values that can be associated with arguments. The idea in using value-based argumentation was to have attacks between arguments failing or succeeding based on the importance of certain values that are referenced by conflicting arguments. Traditionally, computational argumentation has been a primarily formal field of study, but recently, its potential for facilitating human–machine interaction has led to increasingly applied for work, notably in the context of explainable AI (Čyras et al., 2021; Vassiliades et al., 2021) and persuasive technologies (Hadoux et al., 2018; Donadello et al., 2022). Beyond that, researchers have started to ask foundational questions about the integration of formal argumentation with cognitive perspectives, e.g., to study to what extent non-experts find the behavior of different abstract argumentation semantics intuitive (Guillaume et al., 2022) and to model “extra-logical” cognitive reasoning (i.e., reasoning that may be considered irrational from a classical logic point of view) using formal means (Dietz and Kakas, 2021).

Although there are plenty of examples of behavior change support applications, few apply computational argumentation frameworks as a foundation for organizing motives in favor and against what to do to promote health, and in the reasoning in deliberative dialogues between the human and a digital agent. Among the few examples that have used argumentation frameworks for behavior change, an early example in the nutrition domain is provided by Grasso et al. (2000), who explored dialectical argumentation embedding the transtheoretical model of change (TTM) (Prochaska et al., 2015). De Boni et al. (2006) used argumentation through a therapy system in order to change behavior in exercise. Their goal was to apply their system to a specific issue in exercise behavior and to assess the automation capabilities of this system in future studies by improving the argumentation capabilities of the system through personalizing the language used while conversing with the client. Baskar et al. (2017) explored multipurpose argument-based dialogues through a team of agents taking on different roles pursuing different goals in order to address an individual's various sometimes conflicting motives. Roles and an agent's arguments were connected to *argumentation schemes* (Walton et al., 2008), to provide weight on how reliable the argument may be based on the source of the argument.

Chalaguine et al. (2019) and Hadoux and Hunter (2019) investigated how the concerns of the users affect the strength of arguments in dialogue, similar to Baskar et al. (2017). For instance, a user who is not too interested in, say, quitting smoking might become interested if the persuader suggests improvements that quitting can bring out in other aspects of life that the user is more inclined toward, such as social relations and physical activity. Some individuals are more predisposed to act based on their values rather than persuasion through facts (Chalaguine et al., 2019). Atkinson and Wyner (2013) define values as “social interests that a person/agent wishes to promote.” Values are relatively scalable to other values and are important for digital companions in helping a user achieve their goals because values describe desirable goals people want to achieve (van der Weide, 2011). In fact, Perelman and Olbrechts-Tyteca (1969) outlined how people do not use facts but rather their opponents' values and opinions to justify their argument.

The complementary roles of a team of digital coaches to support an individual were outlined by Baskar et al. (2017) for the purpose of managing potentially conflicting motives and needs. A similar approach is presented by Kantharaju et al. (2019); the authors integrate argumentation in a virtual multi-coach platform, in which a group of multiple coaches with their own respective field of expertise and behaviors jointly try to promote healthy behavior in a user. In their study, the authors relate their work to the argumentation schemes *Argument from Expert Opinion* (Walton et al., 2008), and their method of presenting these arguments is implemented through a dialogue game building platform. Some key challenges are listed such as differences in users and how their multi-coach platform can overcome disagreements between the virtual coaches themselves. Kantharaju et al. (2019) also delve into the usage of persuasive social agents for behavior change and which action should be taken by the virtual coaches based on success or failure in abstract argumentation.

Another approach undertaken was by Nguyen and Masthoff where they directed their focus on the effectiveness of motivational interviewing (MI) as opposed to argumentation to persuade the users in their study (Nguyen and Masthoff, 2008). They found that, in some instances, MI is more persuasive than argumentation and that the difference between tailored and non-tailored persuasive dialogue systems are negligible. Miller and Rollnick (2012) described MI as “using a person's own reasons for change within an atmosphere of acceptance and compassion.” The use of MI was also studied by Hörnsten et al. (2014), where the primary healthcare nurses use MI during their health dialogues with patients in order to have a richer and empathy building communication. Hörnsten et al. (2014) conducted 10 interviews with the primary healthcare nurses in the VIP and studied their strategies in their dialogues. Several main themes arose after the interviews, such as “guiding vs. pressuring patients,” “adjusting vs. directing the conversation with the patients” to “inspiring confidence vs. instilling fear.” It is concluded in their study that patient-centered care is preferable, and one key finding in the study is that ideal consultations between the nurse and the patient require empowering words, whereas consultations that include a non-willing patient for behavior change might necessitate pressure, demands for responsibility and challenge.

The need for both supportive and challenging arguments for increasing motivation suggests that a *bi-polar* argumentation framework is suitable to capture both the aspects of challenging the human to change behavior using arguments on the one hand, while also embedding the advantages of MI's sense of acceptance and compassion on the other hand. A bi-polar argumentation framework embeds both arguments in favor and against, for instance, an activity to be conducted (Amgoud et al., 2008). Furthermore, embedding values representing the strength of an argument would allow for comparing arguments (Bench-Capon, 2002). While the atmosphere of acceptance and compassion may be promoted by providing supporting arguments, an emotional parameter expressed as friendliness or empathy is typically expected in inter-human dialogues and has been shown to be also expected in human–robot dialogues, e.g., by Tewari and Lindgren (2022).

To summarize, one of the challenges of this study is to acknowledge the ethical concerns related to evoking cognitive dissonance and potential fear in the individual when challenging their unhealthy choices on the one hand, and on the other hand, providing acceptance and compassion as in MI. The unavoidable human emotional component of arguments and argumentation relating to an individual's choices affecting health is in the following addressed by eliciting the user's preferences regarding the agent's behavior. These preferences are treated as agreements between the user and the agent on how the user expects the agent to perform argument-based dialogues and can be considered a kind of social norm.

## 3. Methods

The research presented in this article applies a constructive, participatory design methodology, and a mixed-methods approach combining qualitative and quantitative research methods. The research was conducted through the following steps:

1. Study 1: Purposed to study perceptions of behavior change in five domains and of digital companions as social actors and collaborators promoting health (40 participated, aged 29–60, see Section 3.1). Based on the results, a framework for designing agent roles and behavior was developed, and a set of argument-based dialogue scenarios were built;
2. Study 2: Extended Study 1 to explore readiness for change in relation to agent roles and behaviors, and perceptions of agent behavior based on the framework (82 participated, aged 29–60). Based on the results a prototype was further developed containing adjusted argument-based dialogue scenarios and a method for tailoring the agent's behavior and roles; and
3. Study 3: Purposed to evaluate the results from studies 1 and 2 in a formative user study of the prototype involving nine experts (public health, nutrition, epidemiology, nursing, and ethnology): The framework, adaptation methods and argument-based dialogues were introduced, evaluated, and further developed.

For data collection in study 1, a questionnaire was developed and applied in English, which was composed based on a set of baseline assessment questions translated from Swedish, drawn from the prototype applications developed as a part of the research project for behavior change addressing:

- General motives for an activity as value directions: questions about the importance, capability, and satisfaction;
- Areas of activities targeted for behavior change: physical activity, stress, alcohol consumption, and tobacco use; and
- Roles of a digital agent in relation to supporting the change of behavior toward healthier habits.

The data collection in study 2 was also done through a questionnaire, which was again conducted in English, which contained a subset of questionnaire 1, limited to only the domains, physical activity and stress. Questionnaire 2 included, in addition, a set of nine dialogue scenarios between a digital agent and two different tentative users. For each of the dialogues, the participant rated the agent's behavior, and what role or roles they thought it was enacted in the scenario.^1^

The data collected using the questionnaires were analyzed quantitatively to find patterns of preferences among roles and behaviors, and qualitatively using thematic analysis for finding themes among open-ended questions regarding activities/goals, roles, and motivations for the agent's preferred behaviors.

The qualitative and formative user study (study 3) was conducted as a part of a participatory design process of the digital coach application for promoting behavior change, divided into three occasions. Study 3 was conducted in Swedish using the Swedish user interface of the STAR-C application. For the sake of readability of the article, terms from the study have been translated into English. Ten domain experts were invited to participate, and nine participated in total.

Four participated in the initial individual session in which they used the prototype, containing five adapted dialogue scenarios in addition to the baseline questions, functionality allowing them to set short-term and long-term goals with related arguments and motives, and the set of questions for adapting the coach's role and behavior. These questions were revised based on the results from the questionnaire study. The participants were interviewed and observed while using the prototype.

A workshop was organized as the second session, where eight domain experts including the four who participated in individual sessions, participated. They were divided into pairs, where the first four participants were paired with each other to start on the same level of knowledge about the system. They were given the task to select activities as goals for behavior change, along with the motives (arguments) for why they want to change, then setting their preferred role or roles and behaviors of the agent. After this, they conducted five dialogues (same as in the individual session). The pairs were instructed to discuss and reflect on the things they experienced and provided examples of how the dialogues ideally would unfold based on their expertise in supporting behavior change. After the sessions in pairs, aspects were discussed with all eight participants. The participants were asked to take notes during the session and were partially observed.

The results of the second session were used for further modifying dialogues implemented in the dialogue demonstrator, and the new versions were evaluated at a third session in a group with seven domain experts participating, including a ninth expert who had not participated in the earlier sessions. The results were also used for further developing the architecture and the generation of argument-based micro-dialogues.

### 3.1. Participants

A total of 40 anonymous participants located in Scandinavia were recruited in study 1 through the Prolific service, and 82 participants in study 2, and 122 participants in total. There was an even gender distribution (58 women, 61 men, and three other) among the participants. The participants' age range was between 29 and 60 years (for age distribution, see Table 1). The age range was chosen based on the most prevalent in stress rehabilitation clinics and the age groups participating in the VIP.

**Table 1:** Age of participants.

**Age**	**Study 1**	**Study 2**	**Study 3**
Below 30	1 (2%)	1 (1%)	0
30–39	21 (53%)	60 (74%)	0
40–49	12 (30%)	11 (14%)	4
50+	6 (15%)	9 (11%)	5
Summary	40	81*	9

*One participant who provided erroneous information about age was excluded in the overview.

Study 3 was conducted as a part of the participatory design process employed in the research program STAR-C, and engaged nine participants (three women and six men) who had been contributing to earlier versions of the prototype in three different sessions (four participated in session 1, eight in session 2, and seven in session 3). The participants had a broad range of expertise, including epidemiology, public health, nutrition, nursing, social work, and ethnology.

### 3.2. Role and behavior of the digital agent

We defined and exemplified four roles that the participants could relate to and choose from in studies 1 and 2. They could also suggest other roles if the roles proposed did not fit their needs. The participants were asked what role or roles they envisioned digital support could take on among the following:

1. *An assistant* that keeps track of your information and reminds you about what you want to be reminded about;
2. *A coach*, similar to a personal trainer who challenges and encourages you to do things;
3. A kind of *health expert*, which informs about the current state of knowledge and gives advice; and
4. *A companion* that is more like a friend, keeping you company and is on your side.

The participants were then asked to provide a scenario and motivate the previous answers.

In study 2, the participants could also assign behaviors to their preferred type of coaching agent along the following: *how brief, how fact-based, how challenging, how emphatic*, and *how friendly*. The participants could select a value on a four-item scale ranging between *very* and *not particularly* in the first three, and the scale had a middle value for the last two labeled *neutral*. This way, a participant could choose a value corresponding to “un-friendly” if they found the agent behaving this way.

After the participants had provided their own wishes for a digital coach, they applied these roles and behaviors to assess the agent's behavior in the argument-based dialogue scenarios.

### 3.3. Framework for adapting the agent's behavior

A framework for adapting the agent's behavior was developed based on study 1 and was further refined based on the subsequent studies. Statements describing the agent's preferred behavior and roles were thematically analyzed and clustered into themes of behaviors and roles. As there were differences among the 40 participants, which seemed to relate to which stage they are in the process of changing behavior, more specific questions to categorize a participant into one of the stages of the transtheoretical model of behavior change (TTM) (Prochaska et al., 2015) were added in study 2.

TTM was first introduced by Prochaska and Di Clemente in the late 1970s and was constructed by six stages of behavior change: *Precontemplation, Contemplation, Preparation, Action, Maintenance*, and *Termination*. Persons in the *Precontemplation* stage do not intend on taking action, in our case within the next 3 months. When it comes to people in the *Contemplation* phase of the stages of behavior change, they are ambivalent toward changing their behavior. The *Preparation* stage is where some people are trying to change and have intentions of changing within the next month. *Action* is when the person has been practicing the new behavior for a short period of time, usually between 3 and 6 months. People in the *Maintenance* stage are already motivated and committed to the behavior change and have been doing the activity for longer than 6 months.

**Figure 1:**
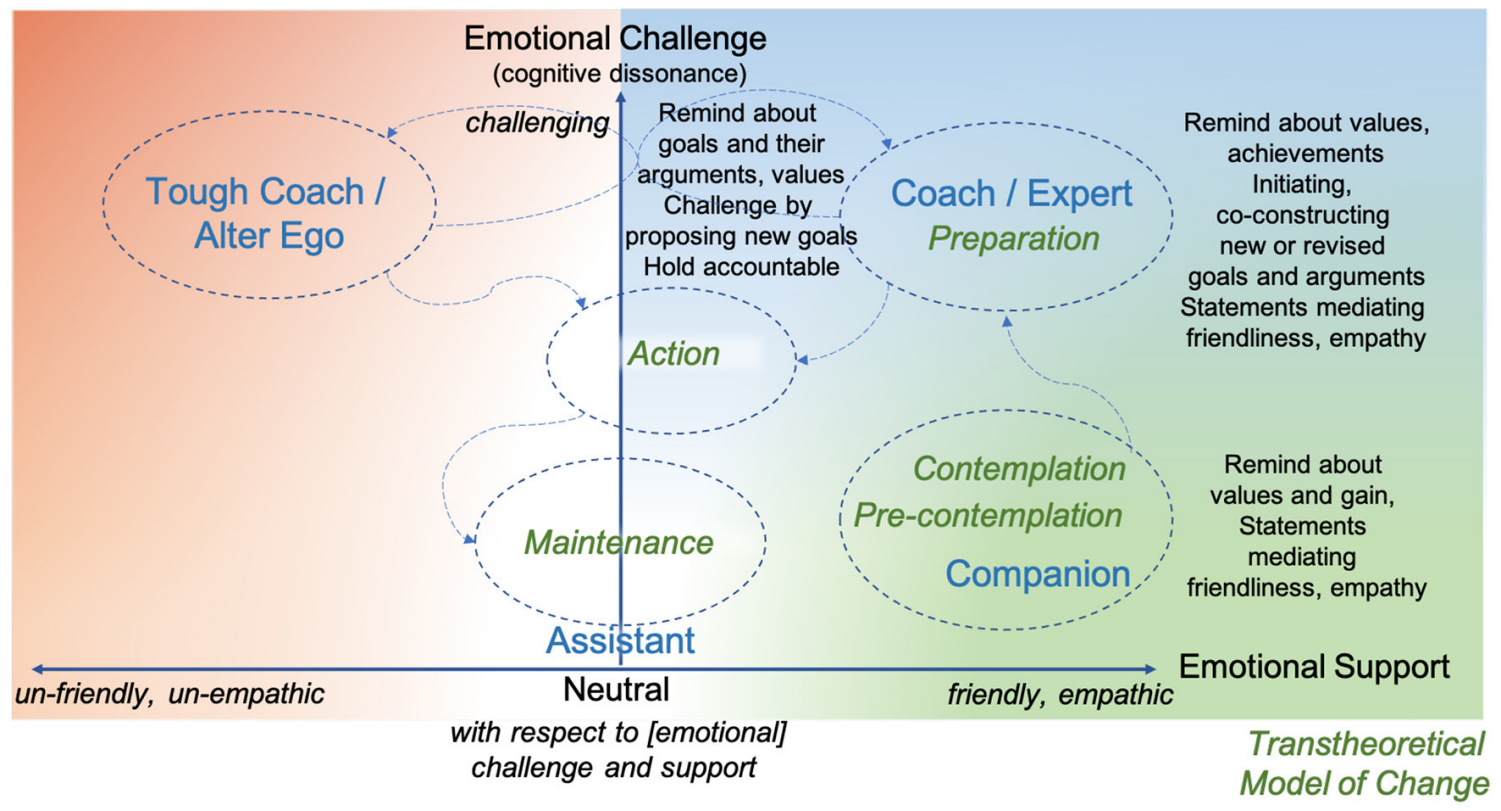
Framework for mapping behaviors of a digital agent along two dimensions: *emotional support* (horizontal) and *emotional challenge* (vertical); to *roles* (blue) and to *stages* in the transtheoretical model for behavior change (TTM) (green), mapped out based on the results of study 1. The framework was applied and evaluated in studies 2 and 3. The arrows represent desirable transitions between TTM stages ending in a stable state of maintenance; there are also potential transitions between roles with a switch to the tough coach and back. The color scheme is inspired by compassion-focused therapy, which uses *green* to represent rest and comfort (soothing), *blue* for energy and action (drive), and *red* as a state of conflict (threat) (Gilbert, 2009). Desired actions provided by participants are exemplified.

The framework is outlined in Figure 1. Some comments provided by participants in the first study are exemplified, along with roles, and stages of change based on two complementary dimensions: One is the extent of empathy and friendliness, and the second is the extent of emotional challenge. This framework was used for designing the nine dialogue scenarios in study 2 and the five scenarios in study 3. An analysis of the data collected in study 2 was conducted for exploring to what extent the choice of agent behavior and role related to what stage of change the participant was in. Furthermore, the roles were further evaluated qualitatively from a user experience perspective in study 3. In the following section, the dialogue scenarios are presented.

#### 3.3.1. Dialogue scenarios in studies 2 and 3

**Figure 2:**
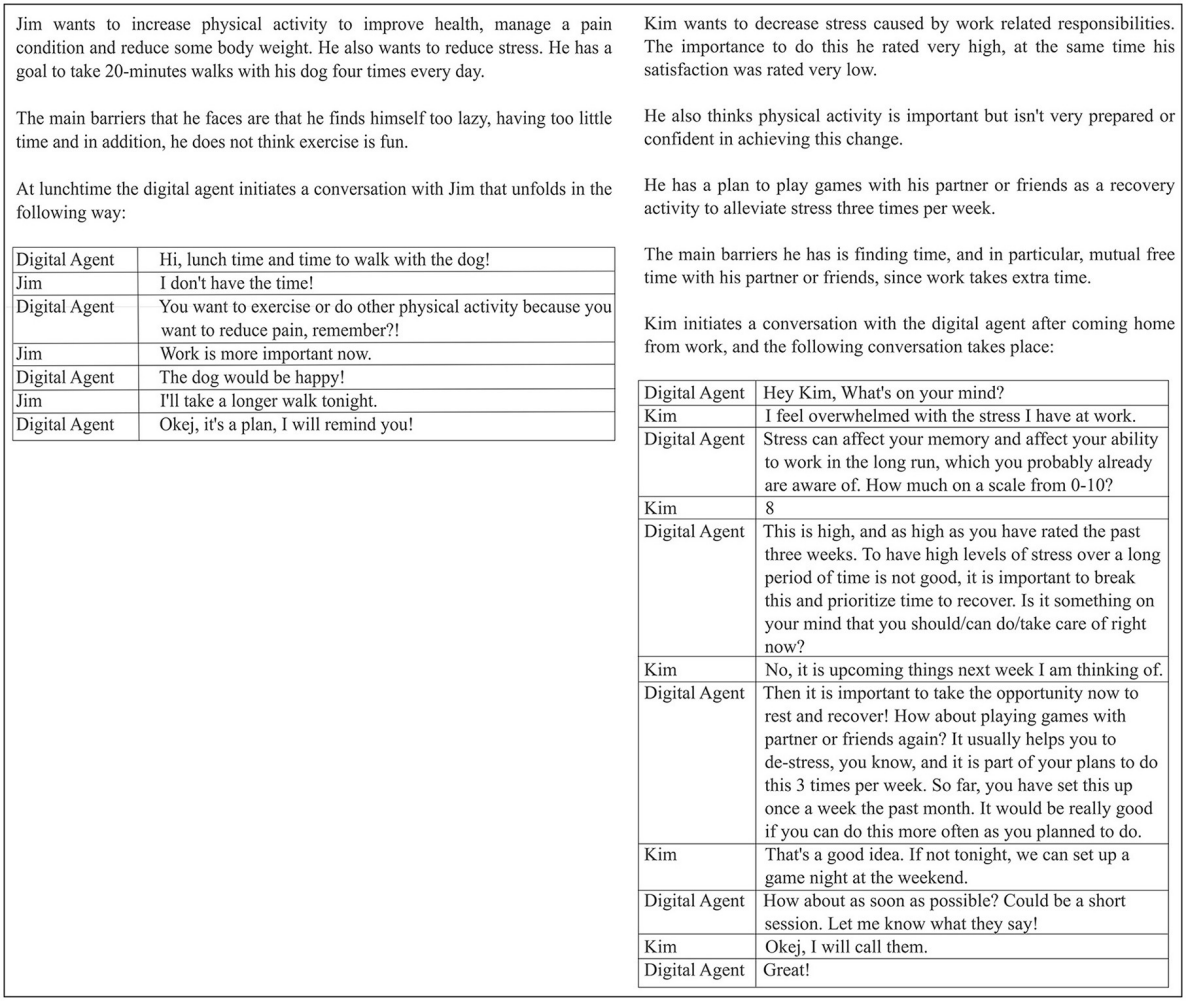
Example of two scenarios: Jim having a short dialogue at lunchtime (1b), and Kim initiating a dialogue at dinner time (2a).

The dialogue scenarios were designed based on the behaviors of preferred coaching agents described by the participants in study 1. The dialogues were engineered with the intent of illustrating how brief, facts-based, challenging, or empathic/friendly an agent can be during the scenarios. Dialogue scenarios containing two characters, Jim and Kim, during different parts of the day/days were authored based on the tentative answers the characters could provide on the baseline questions of the behavior change application, also embedded in studies 1 and 2. The two characters differed, where Jim was more focused on increasing physical activity, and Kim was more focused on managing stress (Figure 2). The nine dialogue scenarios contained between two and 13 statements, 74 in total, with an average of eight statements.

In Example 1b, given in Figure 2, a deliberative dialogue is taking place between the digital agent and user Jim, mutually trying to reach a solution through finding common action. By holding Jim accountable through reminding later in the day and being not completely neutral with respect to emotional challenge and support, the agent portrays characteristics of a brief, superficially friendly, mainly challenging coach (1b in Table 2).

**Table 2:** Scenarios.

**Persona**	**Scenario**	**Time**	**Character**	**Dialogue type**
**Jim**				
	1a	Morning	Neutral assistant	Deliberation
	1b	Lunch	Brief, superficially friendly, mainly challenging coach	Persuasion and deliberation
	1c	Next morning	Friendly, challenging factual expert	Persuasion and deliberation
	1d	Lunch	Non-challenging, brief, friendly and empathic companion	Information-seeking, supportive and deliberation
	1e	Next morning	Non-brief, challenging expert	Persuasion and deliberation
**Kim**				
	2a	Dinner	Non-brief, challenging expert	Persuasion and deliberation
	2b	Next morning	Factual, Friendly and empathic companion	Information-seeking, supportive, and deliberation
	2c	Dinner	Factual neutral assistant wrt emotional support	Information-seeking, supportive, and persuasion
	2d	Next morning	Brief coach, challenging by goal-reminders, and holding accountable	Information-seeking, deliberation

Different types of argumentation dialogue were assigned to different scenarios while maintaining uniformity with the framework in Figure 1. The dialogue types used in the scenarios are *Information-seeking, Deliberative* (deciding about what to do), and *Persuasive* (changing the attitude or behavior of the other agent), as defined by Walton and Krabbe (1995). We complemented these types with a type suitable for the application in focus, which we call *Supportive* to elicit arguments primarily aimed at providing emotional support embedding empathy.

An outline of the characters and types of dialogues with respect to the scenarios can be seen in Table 2. As can be seen, most dialogues consist of elements from different dialogue types.

**Figure 3:**
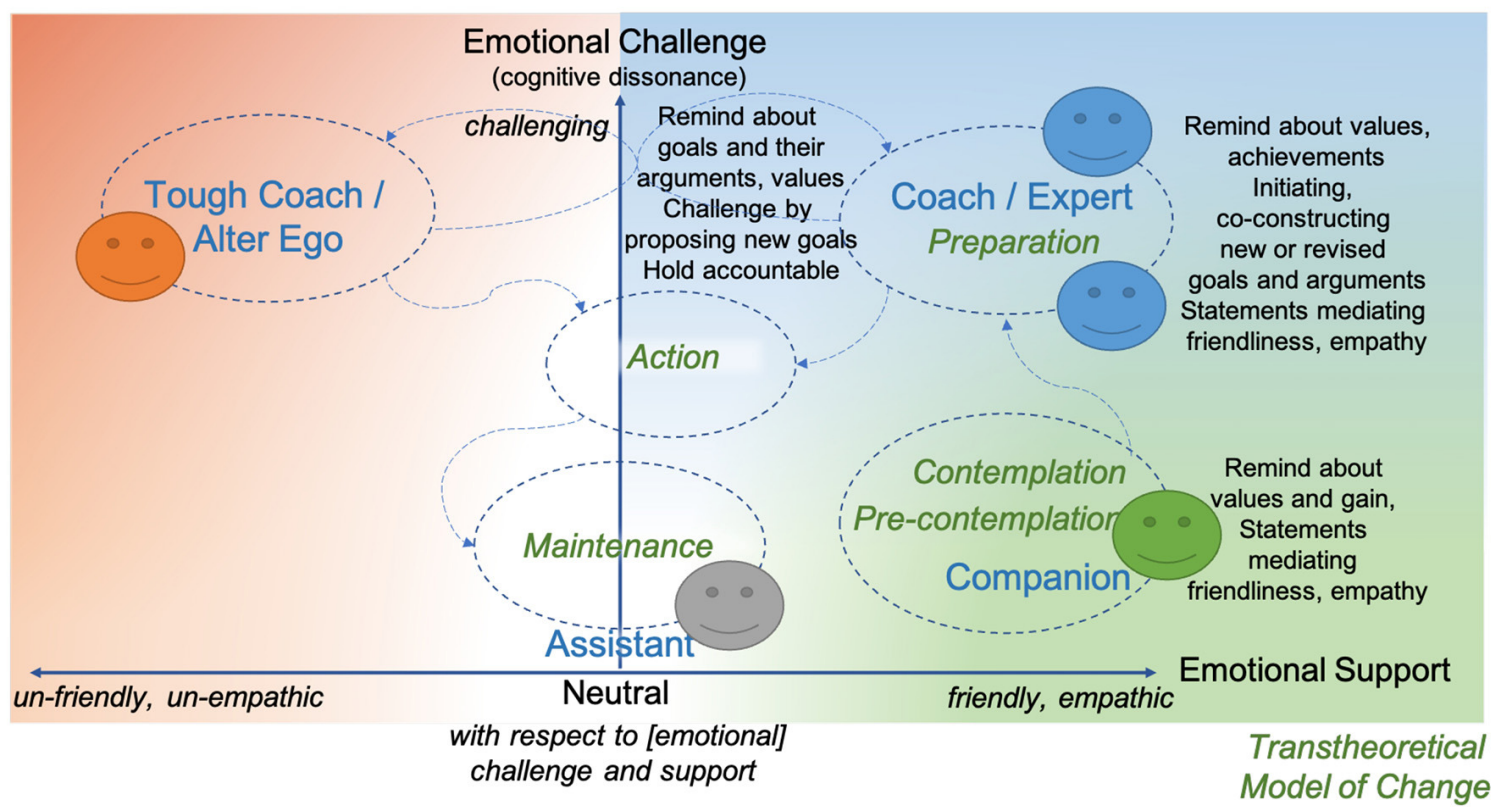
A total of five characters interpreted in the framework for mapping behaviors of a digital agent, Uno is colored green, Dos is gray, Tres and Cuatro are blue, and Cinco is orange. The arrows represent desirable transitions between TTM stages ending in a stable state of maintenance, there are also potential transitions between roles with a switch to the tough coach and back. The color scheme of the agents follows the colors of the compassion-focused therapy as in Figure 1, with the complementary color gray for the neutral assistant.

The five characters applied in the five micro-dialogue scenarios in study 3 were defined based on the model in Figure 1 and on other results of study 2. The characters were named using gender-neutral terms—we chose the Spanish words for numbers (**Table 6**)—and their characters are illustrated in Figure 3.

### 3.4. STAR-C prototype applied in study 3

The prototype applied in study 3 is a mobile application covering the behavior change domains' *physical activity, stress management, nutrition*, and *alcohol and tobacco consumption*. The application contains the following:

- A baseline assessment based on the VIP health assessment consisting of a set of questions, of which a subset was used in studies 1 and 2.
- Goal setting by defining activities to be performed within the coming days/week(s), related to behavior change domains, partly also embedded in studies 1 and 2.
- Setting the roles and behaviors of the digital agent, also embedded in study 2.
- Dialogue demonstrator for evaluating five digital agent characters for the purpose of study 3.

The development of content and structure of the application is done using the content management system ACKTUS, which is a platform for knowledge engineering and design (Lindgren and Yan, 2015). ACKTUS contains a core ontology stored in a graph database (RDF4J^2^) based on the World Health Organization's International Classification of Function, Ability, and Health (ICF),^3^ which is extended with specific and relevant sub-concepts in the class *Personal Factors* and in the class *Activity and Participation*. Other classes are *Body Function and Structures*, and *Environment*, containing social relations and support. ICF is complemented with the class *Diseases and Syndromes* for capturing medical and health conditions.

The ACKTUS ontology also embeds a modified version of the AIF, developed for the purpose to exchange arguments over the web (Chesñevar et al., 2006). An argument (scheme node) is a composite structure consisting of a set of premise nodes (information nodes or *i-nodes*) connected to a conclusion node (i-node) in the graphical database. A premise node relates to information obtained from the user when using the application in the baseline assessment, when setting goals, assessing progress, or in dialogues with the agent. An i-node in ACKTUS is typically linked to a *value*, which can be any that the content modeler decides. Examples of key values in this application supporting behavior change are importance, satisfaction, how fun, how confident, and how prepared a user is to change behavior. Furthermore, the node is also linked to a *concept*, e.g., an *activity* (process) in the Activity and Participation class (e.g., physical activity), or to *objects*, such as body functions and structures, diseases, or relationships. The concept informs about what topic is at focus in a dialogue. In a *deliberation* dialogue, the topic is related to the class Activity and Participation, while in an *inquiry* dialogue, which has the purpose to build new knowledge it relates to a class of objects. Consequently, a conclusion of an argument can be related to an activity (about what to do), an object (about what we know), or an advice.

In ACKTUS, the conclusion node can be of three types: (i) *a decision*, such as in the case of a medical diagnosis, with a value; (ii) *an activity*, in the form of an assessment protocol for what to do next (e.g., a set of follow-up questions); or (iii) *an advice, or piece of information*. These correspond to the argumentation dialogue types mentioned earlier (i) inquiry dialogue; (ii) information seeking or deliberation dialogue; and (iii) persuasive or supportive dialogue. Each composition of premise nodes and a conclusion is associated to an argumentation scheme, which is also modeled in ACKTUS. At the time of conducting study 3, all arguments were associated with the scheme *argument from expert opinion* since the application at that point contained only knowledge engineered by medical domain experts.

The dialogue demonstrator contained a short description of the Jim scenario, on which the five characters' dialogues were built. The dialogues were modeled using ACKTUS. In the initial step, the user was given three answering alternatives: *positive, neutral*, and *negative* for each statement provided by the agent. The next statement posed by the agent depended on the response made by the user. The participants were instructed to select the response based on how they experienced the statement, e.g., liked the statement, or agreed with the statement, or not. Focus was on their experiences and on exploring different ways to respond to the agent's behavior, role, and attitude. Based on the participating domain experts suggestions, the dialogues were modified to encompassing different kinds of responses, which were evaluated by domain experts in a third session.

## 4. Results

The results are organized as follows. In the following section, the readiness levels based on TTM assessed in study 2 are summarized, and the participants' views on motives and barriers for changing behavior. The participants' own expectations of a digital coach or companion in terms of roles and behaviors, and their relation to TTM levels summarized in Section 4.2. The participants' perceptions of the exemplified agents taking on roles and behaviors in the scenarios are presented in section 4.3.

The results from the three studies feed into ongoing work on further developing the architecture and argumentation process for generating person-tailored argument-based micro-dialogues. The argumentation process is introduced and exemplified in section 4.4.

### 4.1. Participants' view on motives for changing behavior related to physical activity and stress

Among the 82 participants in study 2, 19% had always been physically active, and 24% had always been able to manage their stress levels. We consider these being in the maintenance stage of the TTM model (Table 3). For physical activity, a vast majority (75%) is considering changing their behavior within the coming month or within 3 months. A difference is seen in changing behavior to reduce stress, where 30% is planning to make a change. While 23% have a good balance for managing stress, and another 20% has no plans for change coming 3 months, as many as 23% expects an increase in levels of stress (Table 3).

**Table 3:** Study 2 participants' stage of change (TTM), related to what role they preferred, and summary of all 122 participants' choices of roles.

**TTM Stage**	**Number/Stage**	**Assistant**	**Coach**	**Expert**	**Companion 16.5pt**
**Physical activity**	***n*** **= 82**				
**Precontemplation**					
No plans for coming 3 months	4 (4.9%)	25%	0%	50%	0%
**Contemplation**					
Plan to change within 3 months	32 (39%)	69%	50%	34%	28%
**Preparation**					
Plan to change within 4 weeks	30 (36.6%)	67%	63%	53%	20%
**Action**					
Have started to change	N/A	N/A	N/A	N/A	N/A
**Maintenance**					
Change since more than 6 months	15 (18.3%)	40%	60%	27%	20% 16.5pt
**Stress**	***n*** **= 82**				
Precontemplation	17 (20.1%)	50%	31%	31%	19%
Contemplation	9 (11%)	44%	67%	22%	22%
Preparation	21 (25.6%)	67%	67%	33%	29%
Action	N/A	N/A	N/A	N/A	N/A
Maintenance	19 (23.2%)	63%	58%	58%	21%
Termination-risk for relapse	19 (23.2%)	69%	56%	44%	25%
**All in study 1 and 2**	*n* = 122	73 (61%)	68 (57%)	45 (39%)	28 (23%)

The participants' motives relating to a value direction serve as arguments on the needs level of human activity, which is connected to an activity set as goal in the studies (Table 4). The motives were crossing over the two domains for behavior change, such as physical activity was motivated for some as recovery activity from stress which was noticeable in how the participants defined other reasons than those suggested. Furthermore, arguments motivating the choice of value direction, as well as barriers, are captured (Table 5).

**Table 4:** Motivations in terms of value directions (*vd*) for the participant's chosen baby-step activity to increase physical activity or decrease stress.

**I do the activity because**	**Physical activity** ***n* = 122**	**Stress** ***n* = 122**
*vd*_1_. It gives energy	68 (56%)	50 (41%)
*vd*_2_. It's fun. entertaining	47 (38%)	77 (63%)
*vd*_3_. Rest and recover	29 (34%)	91 (75%)
*vd*_4_. Others' expectations	10 (8.2%)	3 (2.5%)
*vd*_5_. Obligations	15 (12.3%)	3 (2.5%)
*vd*_6_. Improve physical wellbeing	101 (83%)	44 (36%)
*vd*_7_. Nurture relationships with immediate family	10 (8.2%)	27 (22%)
*vd*_8_. Nurture relationships with friends and social network	16 (13%)	14 (11.5%)
*vd*_9_. Keep up with society	7 (5.7%)	8 (6.5%)
*vd*_10_. Improve emotional wellbeing	76 (62%)	64 52%)
*vd*_11_. Other: improve appearance. feel more comfortable. escapism. investment in physical and mental health	6 (5%)	4 (3.3%)

**Table 5:** Participants' arguments in favor and against changing behavior to increase physical activity.

**I want to exercise or do other physical activity because**	***n* = 122**	**Type of motivator**
*m*_1_. I want to improve my health	114 (93%)	Introjected regulation
*m*_2_. Research shows that physical activity prevents many diseases	71 (58%)	Introjected regulation
*m*_3_. I want to reduce pain	35 (29%)	Introjected regulation
*m*_4_. It is relaxing	30 (25%)	Intrinsic motivation
*m*_5_. It makes me feel good	81 (66%)	Intrinsic motivation
*m*_6_. It gives energy	68 (56%)	Identified regulation
*m*_7_. It is a social thing	11 (9%)	Identified regulation
*m*_8_. I have to because I sit still all day at work	35 (29%)	Introjected regulation
*m*_9_. I have always done it, it is a habit	10 (8,2%)	A-motivation
*m*_10_. Other: reduce weight (3), kids to be active, reduce stress, improve cognition, mental health, sense of accomplishment, feel stronger, treat physical condition	13 (11%)	Misc
**I don't exercise/or do physical activity because**		**Type of barrier**
*b*_1_. I have never done it regularly, it is not a habit	44 (36%)	Personal: habitual
*b*_2_. I cannot find the time for it	44 (36%)	Personal: organizational
*b*_3_. I do not think that it is fun	34 (28%)	Personal: emotional
*b*_4_. I have too much pain, or other physical condition that stops me	26 (21%)	Physical
*b*_5_. The weather is not good	31 (25%)	Environmental
*b*_6_. It is too expensive to do the things I want to do	15 (12,3%)	Socio-economic
*b*_7_. I would like to do it with others, who are not available	17 (14%)	Social
*b*_8_. Other: depression (2), not enough energy (2), lack of discipline, long distance, fear of falling, others' judgment, laziness, have a baby	16 (13%)	Misc

A low proportion of the participants chose social motivators for their chosen baby-step activity to increase physical activity, social motivators being others' expectations, keeping up with society, and nurturing relationships with friends and family (Table 4). A similar pattern is seen for the baby-step activity to reduce stress, where nurturing relationships with immediate family motivated 22% of the participants. An interesting observation is that the participants seem to have chosen baby-step activities that they find being fun and/or entertaining to a large extent for mitigating stress (63%).

When analyzing the motivators based on gender for their chosen baby-step activity, the answers given were similar in the amount of male and female participants in physical activity as well as for stress. The most apparent reasons for doing their chosen physical activity were physical wellbeing (79% of women and 85% of men), emotional wellbeing (59% of women and 69% of men), and it gives energy (62% of women and 52% of men).

### 4.2. Expectations related to the digital coach's role and behaviors in dialogues

The participants in studies 1 and 2 were asked what role or roles they envisioned digital support could take on among the following (proportion of participants in parentheses) (Table 3): (i) an *assistant* (61%), (ii) a *coach* (57%), (iii) a kind of *health expert* (39%), and (iv) a *companion* (23%), and two participants preferred it to not having a role at all.

**Figure 4:**
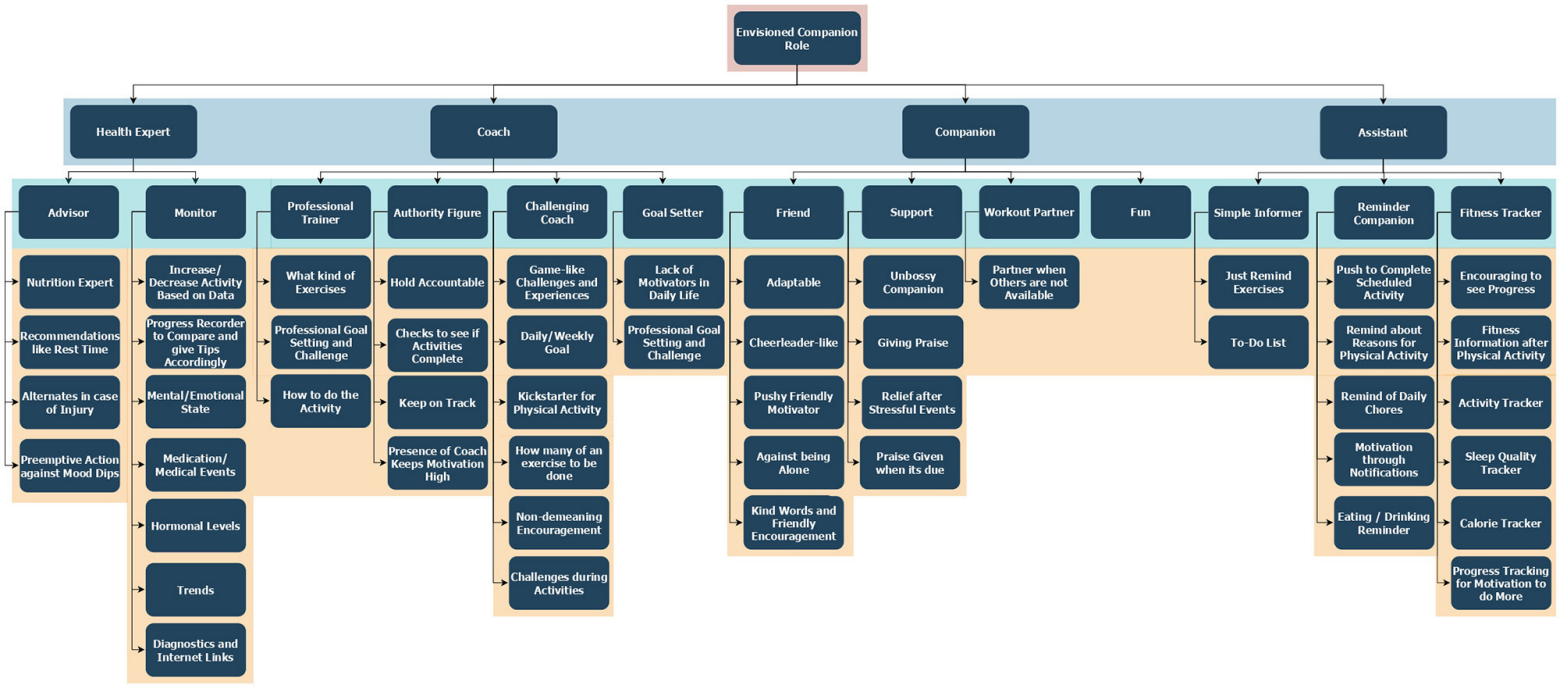
Resulting themes based on study 2 participants' views on the agent's behavior and roles. The blue layer outlines the envisioned companion roles participants have described, whereas the turquoise layer describes the sub-roles the companion can play. The orange layer describes reasons for choosing a sub-role or actions participants would want a companion to execute.

The participants were also asked to provide a scenario and motivate the previous answers. An overview of the themes that emerged is shown in Figure 4. Two major purposes emerged that related to either the digital companion more as a neutral assistant or health expert, or as an engaging coach or companion.

The digital assistant would help track and summarize accomplishments and failures and provide reminders for the person to adhere to their goals. This was also perceived as task for a digital coach. The digital assistant was viewed mostly in comparison to a fitness tracker that is available through smartwatches and mobile applications in the market today. The three main themes that appear under the digital assistant umbrella are *simple informer, reminder companion*, and *fitness tracker*. Uses for the digital assistant in the views of the participants were activities related to such as tracking of sleep and calories but also informing and reminding of the to-dos. Although few similar expectations were summarized under the digital coach and the digital assistant roles, variance of participants' expectations between the two roles is clearly apparent. The digital coach themes were *challenging coach, authority figure, professional trainer*, and *goal-setter*, and it was expected to hold the participant accountable and keep its user on track toward his/her goal through challenge and encouragement. Some participants also wanted the digital coach to embed steps on how to conduct certain tailored physical activities depending on the user's situation.

As for the digital health expert, it would provide personally relevant information and new knowledge, including fearful facts about the consequences if changes are not made to improve health. The main themes that appear in a digital health expert are *advisor* and *monitor* of health status and diagnostics. The *advisor* health expert, in views of the participants, would apprise and recommend for, for instance, preemptive actions against mood dips and adapt to the needs of the user's status related to injury and rest time.

The other categories of purposes related to personal and emotional support are then delivered by a digital coach or companion. Purposes include keeping company, encouragement, motivation, giving inspiration, maintaining reasonable expectations, maintaining discipline, challenge, holding one accountable, telling what to do, and pushing to do activities. Moreover, it could add some fun.

The digital companion role mostly encompassed emotional support and company. The companion was envisioned to be a relief from stressful events and a replacement for human partners in the case of them not being available. The participants also expected the digital companion to be adaptable and unbossy while maintaining its pushy-friendly behaviors.

Furthermore, the relationship between the stages of the TTM and preferred roles (*assistant, health expert, coach, and companion*) and behaviors (*how brief, how fact-based, how challenging, how emphatic, and how friendly*) was explored. This was done to see if the preference for a certain type of behavior or role was dependent on the stages of change (Table 3).

A combination of roles was selected by 56%. The most frequently selected role was assistant (61%) and coach (57%), the expert role was selected by 39%, and the least frequently selected was the companion (23%). The assistant role was less preferred by people in the contemplation stage for managing stress, and people in the maintenance stage for physical activity, compared to how often the role was selected by people in other stages. The companion role seemed to be slightly more interesting to people in the contemplation stage for physical activity, and in the preparation stage for managing stress than compared to people in other stages. Moreover, people rating high importance to change behavior to decrease stress preferred a digital companion over other roles.

**Figure 5:**
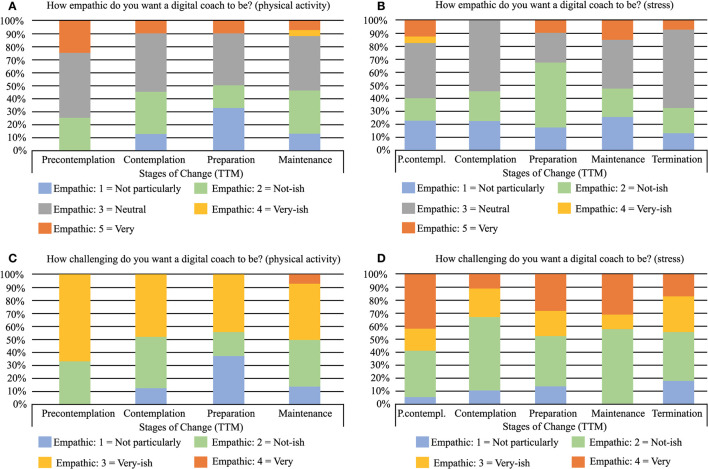
Preferred behaviors of a digital coach, for the different stages of the TTM, for physical activity **(A, C)** and stress **(B, D)**. To be noted: For physical activity, four were in the precontemplation stage, and only one participant was in the termination stage and was therefore omitted in the overview, see Table 3.

Figure 5 shows how the preference for empathetic and challenging behavior is distributed over the stages of change. Approximately 10% across the stages wished the agent to be very empathic, while between 40 and 60% wished it to be not particularly empathic (Figure 5). The rest desired a neutral digital agent, with respect to empathy. About half of the participants wanted the agent to be challenging to a different extent, half to not be particularly challenging. A difference was seen between physical activity and stress, in which participants who wanted the agent to be challenging leaned more toward preferring the agent to be more challenging when supporting behaviors relating to stress than physical activity.

### 4.3. Participants' perceptions of the agents' behaviors and roles

The results of study 2 showed that the participants, in some cases, perceived the agent to express more empathy and friendliness than what they were designed to express, which was the main discrepancy in the cases, the participants had a different perspective on characters and roles (characters in scenarios 1e, 2a, and 2c in Table 6). Due to this, the subsequent characters in study 3 were designed to express more clearly friendliness/empathy, neutrality, and absence thereof (“non-friendliness/non-empathy”), respectively.

**Table 6:** Comparison of defined and perceived character and roles.

**Char.** **(Scen.)**	**Defined character**	**Perceived character**	**Comment**
**Study 2**			
(1a)	Neutral assistant	Also bit friendly/empathic	Agreement
(1b)	Bit friendly, challenging coach	Same	Agreement
(1c)	Friendly, challenging expert	Also coach	Agreement
(1d)	Non-challenging, friendly/empathic companion	Same	Agreement
(1e)	Challenging expert	Neutral coach	Difference*
(2a)	Challenging expert	More coach/companion	Difference*
(2b)	Non-challenging, friendly/empathic companion	Same	Agreement
(2c)	Neutral assistant	More companion	Difference*
(2d)	Challenging coach	Also friendly/empathic	Agreement
**Study 3**			
Uno	Friendly/empathic companion	Empathic, caring, too friendly	Agreement
Dos	Brief neutral assistant	Less empathic, focus numbers	Agreement
Tres	Friendly/empathic, challenging coach/expert	Comforting, safe	Agreement
Cuatro	Friendly/empathic companion/coach/expert	More rehab	Agreement
Cinco	Challenging, non-friendly/empathic coach	Little evil, fun; horrific	Agreement

#### 4.3.1. The participating experts' views

The participants in study 3 reflected on the roles and behaviors of the digital agent in the context of promoting health, while using the prototype application. An overview of their perception of the five example characters is shown in Table 6. While they agreed on the intended characters, roles, and behaviors, what they liked and did not like varied. Uno was preferred by one who found it to be encouraging and “here and now.” The most preferred character was Tres, the empathic and challenging coach/expert, followed by Cinco, the non-friendly and challenging coach. Those who preferred Cinco found it intriguing, “a little evil,” and fun, compared to the other examples, and as a way to “push.” They found it being good that it is straight to the point and good for the memory to be reminded.

Those who liked Tres the most, also disliked Cinco the most, using words like “terrible,” “not acceptable.” One of the participants who preferred Tres and disliked Cinco motivated this by wanting a digital companion or coach who could provide a basic sense of comfort, safety, and trust, which would not work with Cinco. On the other hand, when the basic foundation of trust and comfort is established, the agent could in some moment turn into the Cinco character to provoke/challenge the participant's attitude: “...then it can be ok with more harsh comments as a kick in the butt.” More comments on that a variation in behavior and a mix of attitudes were preferred, both “soft, compassionate but could be firm.”

General comments concerned the amount of information about health in the statements provided by the digital agent. Shorter, to-the-point statements about health were desired; better to be more briefer than too facts-based and lengthy in arguing why changing behavior is desired. Suggestions of dialogue elements included ending with a question that the person can respond to, which also works as a challenge, something to think about.

Alternative ways for the user to respond to arguments were suggested, partly to make the user reflect and collect the user's view on the argument, partly to lead the reasoning process forward toward a positive conclusion about what to do. In addition to information-seeking purposes, the following three general responses were identified:

(i) to state *confirm, reject* (potentially moving forward in time), or *undecided* (expressing ambivalence);
(ii) *confirm, reject*, or *undecided* as in previous but also including a *reason* for this among barriers or motivators identified as relevant to the individual (pose a supporting or attacking argument); or
(iii) to reason about what *emotional support or challenge* the individual needs in the current moment (change topic to how to act).

Examples were embedded in new versions of the five dialogue scenarios and discussed at a follow-up session with the experts. While confirming that their perspectives and suggestions were embedded in the new versions, they also highlighted the cultural aspects concerning *how to* express things in dialogue with different people.

### 4.4. Person-tailored argument-based micro-dialogues

The application STAR-C used in the study is being developed to embed a digital coach, which utilizes value-based argumentation embedding supporting and challenging arguments. When developing the STAR-C coach module further based on the results of the studies presented in this article, we explore how argumentation schemes can be utilized. The STAR-C mobile application uses the knowledge base embedded in the ACKTUS platform (Lindgren and Yan, 2015), as introduced in Section 3.4. The user's information that is collected at baseline and in daily use will be used by the system for tailoring short dialogues (micro-dialogues) to the individual. In this section, a high-level description of the construction, evaluation, and the application of arguments in dialogues with a user is presented. Furthermore, the findings presented from studies are applied in an example case based on one of the participants. The purpose is to exemplify the adaptation of roles and behaviors to the individual's preferences, goals and values, and the argumentation process. Also, the different types of responses in the dialogues are exemplified.

#### 4.4.1. Representing generic knowledge and knowledge about the user

The following is an example of how an argumentation between a digital agent and one of the participant from our study, Jane (alias), could play out based on Jane's value directions, actions, motives, and preferences regarding the digital agent.

*Jane wants to increase physical activity to improve health, which she rates most important, and lose weight. She also wants to reduce stress, which she rates as very important. She has a goal of walking her dog for 30 min per day and has stated to the digital companion that walking her dog is the best method for dealing with stress, as recovery activity, and that she has to do it. Therefore, Jane wants her digital agent to be a companion with some empathy, but also a “Tough Coach/Alter Ego” to challenge her and be pushy at times to support her to reach her goal*.*The main barriers Jane faces is that she lacks energy, thinks exercising is not fun and the weather where she lives is usually bad. Moreover, she often does not have the time*.

At baseline, our example user Jane had assessed what behaviors (*bh*_*i*_) she prioritized to change and selected increasing physical activity (*bh*_1_) and activities to decrease stress (*bh*_2_). For each of these, she assessed *how important, how prepared* she is to make a change, *how confident* she is to succeed, and *how satisfied* she is with the current situation. We will, in the following example, apply only the *importance* value and assume she is in the *preparation* stage of TTM, aiming to take action within the coming weeks. At baseline, she had also assessed what is *motivating* her to change behavior relating to physical activity: *m*_1_ (improve health), *m*_10*a*_ (reduce stress), and *m*_10*b*_ (reduce weight); and *barriers* (i.e., counter arguments) for changing behavior: *b*_2_, *b*_5_, and *b*_8_ (Table 5).

At run-time, when defining an activity meeting a short-term goal, the user selects which behavior the activity aims to change (e.g., too little physical activity and/or stress), what they aim to do (Jane in our example is walking her dog 30 min four times per day) how *important* (value) the activity is and how *fun* she expects it to be (value), and with whom they would like to do the activity with (in our example, Jane selected her pet for her walk with the dog). Furthermore, motives related to value directions (*vd*_*i*_) for taking a walk with the dog are captured (*vd*_3_, *vd*_4_ in Table 4), as well as the *social* parameter with whom or what the activity is planned to be done, which in our example, also tells who may be disappointed if this activity will not be done. The *goal* is set to do the activity for 30 min four times per day.

In addition to person-specific knowledge, the agent has general knowledge applicable in Jane's case, which it can retrieve from its knowledge base (**Figure 8**). General knowledge is formulated as *generic arguments* (*ga*). Each argument is associated with an *argumentation scheme* (*as*). Two schemes defined by Walton et al. (2008) were applied: *argument from expert opinion* (*as*_1_) and *argument from position to know* (*as*_2_), as exemplified as follows:

*ga*_1_ Physical activity increases energy levels (*argument from expert opinion*).
*ga*_2_ Recovery activities are necessary to decrease stress levels (*argument from expert opinion*).
*ga*_3_ Humans and other animals become happy when socializing and unhappy when opportunities are missed socializing (*argument from position to know*).
*ga*_4_ A happy state increases energy and decreases stress levels (*argument from position to know*).
*ga*_5_ Increased energy levels make one a better worker (*argument from position to know*).

The first two statements are asserted to be true by experts in the domain of stress management; subject domain is, in this case, psychology. The following three are generic assumptions from positions to know, which can be seen as examples of statements by a person sharing their own experiences with others. Consequently, arguments associated with the different argumentation schemes are ranked differently reliable for instance, an argument from the expert opinion grounded in relevant clinical experiences can be considered stronger than an argument from position to know (Lindgren and Yan, 2015). However, to an individual, the argument that the dog will be happy may be a more personally relevant and, therefore, stronger argument than one based on expert opinion.

**Figure 6:**
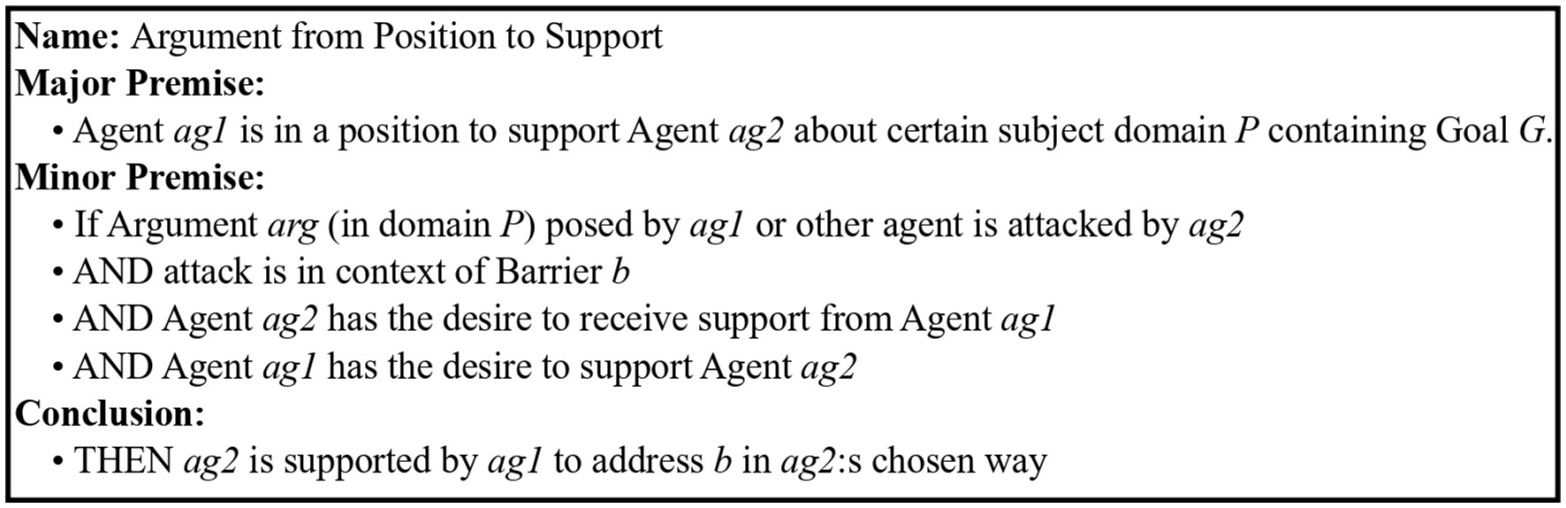
Argument from Position to Support.

**Figure 7:**
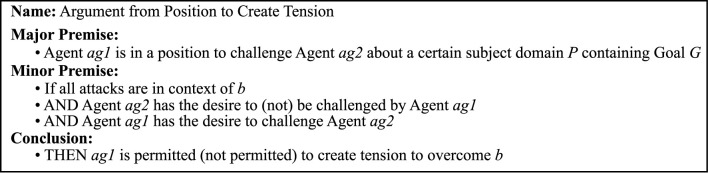
Argument from Position to Create Tension.

The studies presented, in this article, explored argumentation from the additional positions providing emotional *support* for the purpose of providing a sense of being on their side and *challenge*, which may increase cognitive dissonance and tension. These purposes are different from the purposes information seeking, inquiry, deliberation, and persuasion dialogues as defined by Walton et al. (2008). Therefore, to encompass argumentation with purposes other than those defined by Walton et al. (2008), two argumentation schemes were defined: *argument from position to support* (*as*_3_) (Figure 6) and *argument from position to create tension* (*as*_4_) (Figure 7).

A barrier *b* is identified as something preventing the person (*ag*2) from doing a desired activity and can be viewed as an argument for why a person would not pursue his/her goal *G* (Figure 6). In the situation when the person's argument for not doing the intended activity that would pursue the goal (e.g., being too tired to do physical exercise) is questioned (attacked or undercut) by the digital agent or other (e.g., physical activity gives you energy), the agent complying with the argument from the position to support scheme would take the supporting position and state, for example, the following:

*ga*_6_ There are good reasons not to conduct the planned activity targeting the desired goal, so based on the highlighted circumstances; it is better not to do it at this point (*argument from position to support*).

On the other hand, if the agent would instead comply with the argument from position to create tension, knowing that the person wants to be challenged by the agent, then the agent is allowed (permitted) to create tension evoking some cognitive dissonance or other emotional engagement to overcome the barrier. However, if the person has stated that challenging behavior is not desired, the agent is not permitted to create tension even if the agent assesses this to be the best strategy based on other factors. The following is an example:

*ga*_7_ Weather should not prevent people from conducting activities since people are not made of sugar (*argument from position to create tension*).

These argumentation schemes can be used by the agent to adapt its reasoning to a situation, and reason from which position (role and character) the agent takes on expert, coach, companion, and assistant or the challenging alter ego, this is based on a mutual agreement on the social norms to be applied in the dialogue.

#### 4.4.2. Building and using arguments

**Figure 8:**
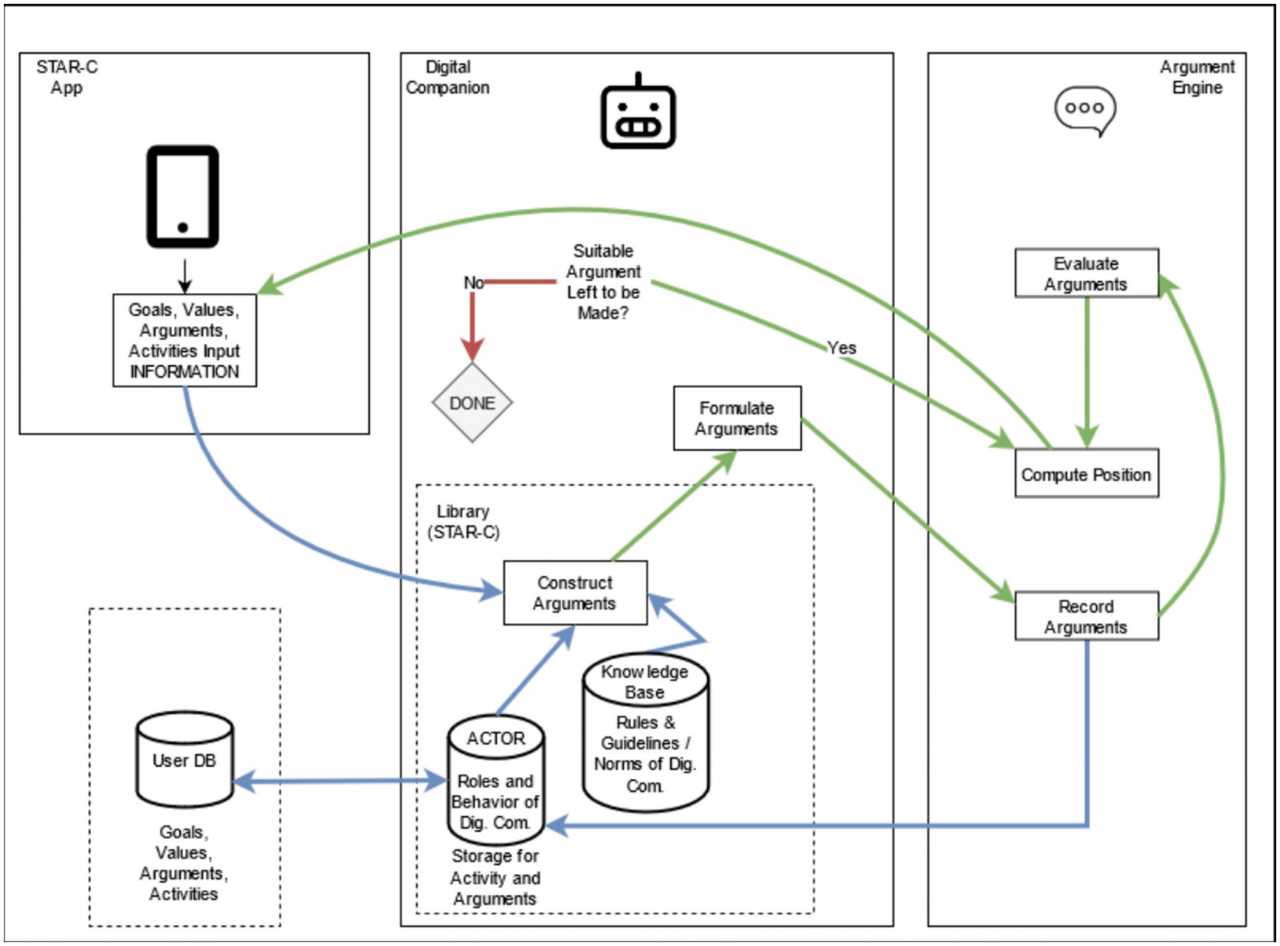
Process of construction, evaluation, and application of arguments. The blue arrows follow the argument to be constructed while the green arrows follow the dialogue path with the user.

The following is a brief overview of the process of constructing and applying arguments in a dialogue, as shown in Figure 8. The approach was inspired by Ballnat and Gordon (2010) argumentation process and the *sufficient condition scheme* based on Walton and Krabbe (1995), which was extended by Atkinson et al. (2006) to embed values. The blue arrows in the figure follow the argument to be constructed. The green arrows follow the path to a dialogue with the user.

When the dialogue is activated by the user or the agent, this triggers the *Construct Arguments* module which fetches the relevant goals, values, activities, and arguments connected to the user. The module puts this information into the relevant contextual information fetched from the *Knowledge Base* confirms adherence to rules and guidelines, and construct arguments utilizing the information. After the construction of the arguments, the *Formulate Arguments* module translates the arguments into a culturally adapted format suitable for a dialogue (e.g., language, language suitable for subgroups in society). The arguments are then recorded with the *Record Arguments* module to be sent into the repository for utilization in future dialogues and arguments.

The arguments, after being recorded in the database, are referred to the *Evaluate Arguments* module to be used in dialogue with the user. The evaluated arguments are then dispatched to the *Compute Position* module. The *Compute Position* module takes on the important duty of combining the behavior and role of the coach, depending on the situation of the user (explained in more detail with examples below) but also is the module which sends the supporting argument or counterargument to be displayed to the user for the continuation of the dialogue. There is always the possibility of the user having something that does not allow them to do the activity suggested or reminded about by the digital companion. The *Argument Left to be Made* component in the digital companion ends the dialogue in a proactive manner, as shown in the dialogue with Jim in Figure 2, if that is the case or when there are no more arguments to be made. If there is room to propose additional supportive arguments or counterarguments into the dialogue with the user, the green arrow dialogue loop continues.

To represent the argumentation-based process in a formalized manner, the extension of Walton's (1996) *sufficient condition scheme* laid out by Atkinson et al. (2006) is adopted as the general scheme for the agent, which can embed arguments from different positions rooted in other argument schemes. Argumentation schemes function as templates for reasoning, in this example, embedding a positive prediction of the effects of performing the activity the user had planned, both on the action and value-direction levels of activity. The scheme in Atkinson et al. (2006) is given as follows:

*as*_5_*: In the current circumstances R, we should perform action A, which will result in new circumstances S, which will realize goal G, which will promote some value V*.

Since contextual knowledge, such as domain knowledge, is essential when reasoning about health, we further extended this scheme regarding current circumstances by specifying different categories of circumstances. In our example, the agent has the following information about Jane's situation, interpreted in terms of the argumentation scheme and available relevant knowledge retrieved from the knowledge base. Relevance is determined by the domain of behavior change and which role the agent is taking on based on the user's preferences and stage of change:

**R: (Current Circumstances)**

- AgentPreferences = (lunch-time is a preferred moment to interact with the agent; empathic, challenging companion);
- Goal = (walk the dog 30 min);
- Motives = (*bh1:* increase physical activity (importance-value: most); *m1:* improve health; *m10a:* reduce stress, *m10b:* reduce weight; for the chosen activity *vd3:* rest and recover; *vd5:* obliged to walk the dog);
- Barriers = (*b8:* may be lacking energy, *b2:* may be lacking time, *b5:* rainy weather);
- GenericKnowledge = (*ga1 - ga7*);

**A: (Actions)** Walk the dog for 30 min**S: (New Circumstances)** More energy, Jane and the dog are happy**G: (Achieved Goals)** Walked the dog for 30 min**V: (Values)** Increased physical activity is most important, reduced stress very important, improved health, reduced weight, and increased energy level.

To continue with our example, at lunch time, the digital companion initiates a dialogue with Jane according to her preferences, with a set of constructed arguments, which are updated during the argument process based on new circumstances provided by the user and with the following set of potential actions, including the activity Jane has specified as the target activity:

1. *Walk Dog 30 min*: The action that follows Jane's plan to increase physical activity,
2. *Walk Dog 15 min*: The action that partially follows Jane's plan to increase physical activity,
3. *Let Dog out in the backyard while having lunch working*: The action that barely follows Jane's plan to increase physical activity but may follow Jane's plan to decrease stress, and
4. *Do Nothing*: The dog is not cared for, so this is not an option due to her obligations.

**Figure 9:**
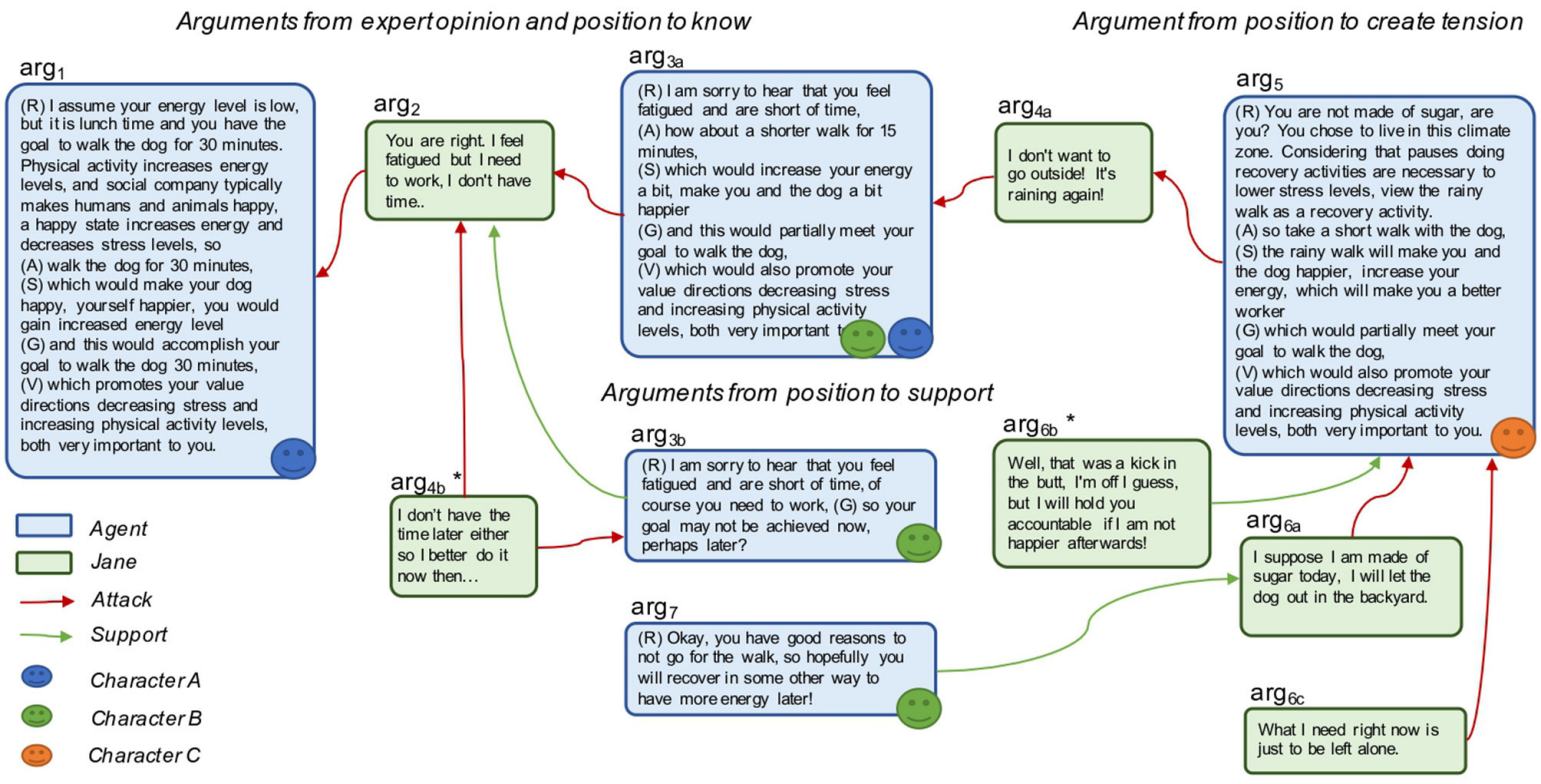
Attacking (challenging) (red arrow) and supporting (green arrow) arguments posed by the agent (blue) and Jane (green). The agent can choose between a supportive argument or a challenging argument as a response to Jane's first argument, which could lead to different outcomes. The arguments arg4b * and arg6b * indicate the desired outcome.

The dialogue is initiated by the agent, based on the argumentation scheme *as*_4_; it poses Argument *arg*_1_ focusing Barrier *b*_8_, see Figure 9 to see how the dialogue could unfold. One decision point is whether to select a more challenging or more supportive attitude in step 3. Since Jane brings up another barrier (Barrier *b*_2_), the agent follows up in the next step, addressing this barrier.

When Jane brings up yet another barrier, the weather condition (Barrier *b*_5_), the digital agent decides to use the harsher counterarguments, adopting the pushy character as per Jane's choice for persuading her to do it and hold her accountable.

Jane has three alternative responses in the example; in the second alternative, Jane picks up on the potential “loving boot effect” (Blakey and Day, 2012), a stimulation that “kicks” Jane to achieve higher performance, leading the agent to follow-up the walk choose the question about how happy she is afterward. The third alternative is an example when Jane may chose to counteract by changing the topic toward what she needs, rather than what to do (Figure 9).

#### 4.4.3. Evaluating and selecting arguments

The agent starts off with selecting a subject domain to target, i.e., topic, based on which assumptions are generated about current circumstances based on the available information and contextual information, such as time of the day.

The order in which the action *A* is selected relates to the potential options that are available to the agent, the user's selected goals and activities, their assessments of importance and accomplishments so far, and the roles and behaviors preferred by the user.

The agent would rank the set of potential actions based on utility in the value functions (importance and physical activeness in this example since increasing physical activity was ranked highest before reducing stress) and to what extent the action would fulfill the user's short-term goal. The agent would then begin with the option with the highest value, then after evaluating the response from the user and potentially revise the list, go down the list until there is a reason to end the dialogue. Based on the responses of the user and the barriers they have, the agent computes position to be supportive or provoking, along with a re-evaluation of the order of actions.

The subject domain is a factor when evaluating arguments from the agent's perspectives as there are multiple domains in which the user might want to change behavior. Therefore, varying roles and behaviors might be necessary for certain domains (e.g., a user might be in one TTM stage for increasing physical activity but might be on a different stage when it comes to reducing stress as in this example), while it might not be of the essence in other domains. One strategy the agent can apply is to broaden the subject domain to include more topics (e.g., in our example, also reducing stress) to strengthen the values of conducting an activity when it could serve more than one goal or value direction.

When the user attacks an argument put forth by the agent, the agent must distinguish the barrier that is holding the user from achieving their goal *G*. This is achieved through the ToM the agent has constructed about the user, in combination with the current situation, e.g., weather conditions and time of the day. The counterarguments presented by the user are saved into the repository to be analyzed for future reference and usage in arguments to come.

## 5. Discussion

The purpose of the research presented, in this article, is to use AI systems to empower individuals to progress in their pursuit of improving health and physical and emotional wellbeing through a change of behavior. This aligns very well with the definition by Nowak et al. (2018) of HCAI as AI that focuses on “collaborating with humans, enhancing their capabilities, and empowering them to better achieve their goals.”

In the notion of *collaboration*, there is a social aspect embedded relating to coordination and agreeing on goals and a division of tasks, typically relating to what roles the actors are enacting. In the studies presented in this article, the digital agent's roles and behaviors as a social actor are explored from the viewpoints of potential users and domain experts, which is discussed in the following section.

Furthermore, when coordinating and agreeing on goals and the division of tasks in an envisioned collaborative journey of the agent teaming up with the user, instruments for the agent to apply are key.

Natural argumentation allowing the user to respond in any way they like would allow the user to express themselves freely and with the language they usually use. However, in this study, structured dialogues are used for the purpose of allowing domain experts to evaluate and verify the agent's behavior, as well as to obtain structured information from the user for feedback and research purposes. The STAR-C application provides some freedom to define their activities and goals, motivators, and barriers, along with the structured alternatives. The structured parameters are embedded to find themes of concerns, activities targeted for behavior change, and for measuring outcomes and trajectories of change from a public health perspective. The purpose is also to generate supporting and challenging arguments based on momentary assessments, as well as analyzes of activities over time.

The exploration of participants' views on roles and behaviors of a digital agent in the context of supporting behavior change for improving health generated the framework for outlining an agent's emotional *support* and *challenge* in relation to the agent's role and the user's stage of change. We exemplify how the agent can take on behaviors and roles and shift between these by using argumentation schemes. To encompass also the emotional support and challenge, two schemes for the purpose were defined to complement the schemes outlined by Walton et al. (2008). We built new schemes for the two and showed their usage through an example. In connection with the two new schemes, two new positions, *Position to Support* and *Position to Create Tension*, were introduced. While support and challenge is embedded in the argumentation frameworks' attack and support relations, there is currently no usage of such argumentation schemes through a multi-charactered digital companion for improving health, as far as we are aware of. This approach allows for managing arguments that have both emotion-based grounds and knowledge-based grounds, for instance, medical knowledge.

Our approach provides means to reason also about the ethical aspects in a dialogue situation which may trigger cognitive dissonance, which in turn, for some individuals, may increase anxiety and stress (Tengland, 2016). Guided by the domain experts' and participants' perspectives, the user's preferences are embedded in the two argumentation schemes as the representation of the mutual agreement on how the collaborative relationship should be actuated in terms of support and challenge creating tension. Furthermore, allowing the user to raise the topic of how to act as the third type of response paves also ways to allow the user to challenge the agent's behavior.

From a foundational argumentation perspective, it is worth highlighting that the results hint at the relevance of “soft” and informal behavioral and interactive properties of argumentation-augmented agents. In particular, our study results indicate that the preferred properties, e.g., regarding how *challenging* an agent is (which can, in our context, be interpreted as how consequent and with which attitude an agent will attempt to persuade with rational arguments), are subjective. Although these observations are not particularly surprising in their preliminary nature, it is worth noting that very little is known about human attitudes regarding the behavior of agents that have been augmented with (formal) argumentative capabilities. Even on object level, when assessing the inference results provided by abstract argumentation semantics, a recent study shows that the expectations of non-expert humans are not aligned with the behavior of many argumentation semantics that is popular in the research community (Guillaume et al., 2022). There seems to be little work that systematically studies how meta-level properties of computational argumentation, such as the way arguments or argumentation-based inferences are rendered to human users by a user interface, affect credibility, persuasiveness, and engagement. Considering the widespread success of choice architecture (Thaler et al., 2013) (also referred to as *nudging*), i.e., the rendering of information in a way that maximizes the intended impact on information consumers, this raises the question whether future approaches to argumentation for human–AI interaction can potentially benefit from fusing formal (“hard”), object-level argumentation with informal (“soft”), meta-level optimization, and personalization.

To summarize, our approach using computational argumentation and argument schemes provides transparency with respect to the agent's roles, behaviors, and sources of its arguments. Future user studies will explore how the user relates to the roles and positions of the agent in situated activities and the agent's support in the pursuit of improved health in these situations.

### 5.1. Participants' perceptions of emotional support vs. challenge

Since the results did not provide clear patterns of preferences among roles and behaviors relating to which TTM stage a user may be in, we choose to rely on the individual user's preferences, together with suggestions provided by the domain experts on how to address individuals in different stages of readiness for change.

An interesting observation was that the participants perceived neutral behavior as friendly and empathic in the situation when the human expressed distress due to overload at work. This occurred when the persona in the scenario shifted from the first one focusing on physical activity to the persona dealing with stress and worries. Their perception of the neutral agent as being empathic and friendly may be due to this kind of behavior is expected in such situations, and consequently, the participants interpret the agent's neutral behavior as such. One could also expect that the participants would have experienced a lack of empathy in this situation, as some participants expressed in a study on humans interacting with a robot (Tewari and Lindgren, 2022). However, as argued by Pulman (2010): “... a Companion which behaved in the same way whatever our emotional state would be thought of as insufficiently aware of us. But this may not mean that the Companion itself has to express emotions: all that is necessary to achieve this is the ability to recognize our own displays of emotion.”

In the three cases when there was a difference between the intended character and behavior and how the participants rated the agent's behavior, the difference mainly consisted in that the participants rated the agent's empathy and friendliness higher than was intended, which also led to classifying these agents being companions to a larger extent. This we interpret as a cultural aspect; the participants were located in Scandinavia, where the way to express empathy and friendliness may differ from other places, a phenomenon which has been recently studied from an affective agents' perspective (Taverner et al., 2020). We plan to broaden our subsequent studies to include participants of various backgrounds to test our interpretation's validity.

People rate the high importance of changing behavior to decrease stress and tended to prefer a digital companion over other roles. This aligns with the expectation of a more empathic response in the exemplified dialogue on managing stress.

An outcome from the responses obtained from the participants for the question of which agent role they preferred in studies 1 and 2 was that more than 75% of them did not choose the companion role. On the other hand, the domain experts, although few, who experienced the dialogues with the digital agent through the prototype preferred the friendly and empathic role more than the other roles. The participants in studies 1 and 2 answered this question before they had encountered the scenarios and may have had a different view after evaluating the scenarios or if they had experienced the dialogues as the participating experts did through the prototype. Future studies will provide hands-on experiences of the different roles, which is expected to provide more reliable results.

The group of participants contained a large proportion of 30–39-year-old people in studies 1 and 2. It would be interesting to further analyze the data to explore whether the preferences that the group as a whole differ when studying the aspects from the perspective of age groups.

Studies on preferences regarding agent characters have shown that age is a deciding factor when it comes to choosing a digital companion. For instance, in Hurmuz et al. (2022), older adults preferred personalized content when interacting with a digital companion. Furthermore, when looking at the features of a digital companion in terms of friendliness, expertise, reliability, authority, and involvement, the general and elderly population preferred a gendered digital companion, specifically a young female (ter Stal et al., 2019). As for the type of messages, users would like to receive from such technologies, it has been found that reports about progress, sent at the right time, rather than something educational, is preferable (Klaassen et al., 2013). It is important to highlight, however, that there is currently a lack of studies on the preferences of roles and behaviors of digital companions in the domain of behavior change. Our ongoing and future study includes extending and implementing tailored dialogue capabilities of the digital companion. User studies will be conducted to further explore how participants in different stages of change and with different preferences relate to the digital agent in real-life settings. Furthermore, the effects of having argument-based dialogues with the digital companion on users' attitudes toward and actual changes of behavior, as well as wellbeing, will be studied in a randomized control trial over 6 months and continued use additional 6 months.

## 6. Conclusion

The studies presented in this article have explored the roles that digital companions can play in supporting behavior changes, and the attitudes that users, as well as domain experts from different disciplines, have toward them. A focus was placed on argumentative approaches, both conceptually, i.e., expectations and perceptions regarding the argumentation-related behavior and interaction, and practically, in the forms of argumentation-based system architecture and an early-stage prototype. The findings provide initial quantitative and qualitative insights that highlight the importance of “soft” non-formal behavioral aspects of argumentation-augmented agents in human–AI interaction scenarios but also indicate that some of the desirable properties of these aspects can be subjective and context-dependent.

Assuming that a major purpose of computational argumentation is the facilitation of human–machine interaction, we hence conclude that a nascent, high-potential research focus of the human-centered AI community in general, and the argumentation community in particular, could be the integration of “rational” argumentation-based reasoning by computational means with human-centered approaches regarding the presentation of arguments and argumentation-based inference results. To advance this research direction, results and methods from adjacent disciplines, such as behavioral economics and psychology, need to be incorporated. In turn, these disciplines can potentially—given that such an integration succeeds—benefit from the computational tools that the argumentation community provides.

## Data availability statement

The original contributions presented in the study are included in the article/Supplementary material, further inquiries can be directed to the corresponding author.

## Ethics statement

The project was reviewed and approved by the Swedish Ethical Review Authority (Dnr: 2019-02924 and Dnr: 2020-02985). The participants provided their written informed consent to participate in this study.

## Author contributions

HL, KK, and SW: idea development and studies 1 and 2. KK: led the authoring, reviewed related work, and major work on the results relating to the person-tailored argument-based micro-dialogues. KK and HL: study 3 and development of the agent dialogue demonstrator. HL, SW, and TK: edit and review. HL: initial ideas, an overall responsibility of studies, the ACKTUS platform, and STAR-C application. All authors contributed to the article and approved the submitted version.

## Footnotes

^1^The two questionnaires are found in the Supplementary material.

^2^ https://rdf4j.org/

^3^ https://www.who.int/standards/classifications/international-classification-of-functioning-disability-and-health
