# Supplementary material for: Argument-based human–AI collaboration for supporting behavior change to improve health
Source: Front Artif Intell. 2023 Feb 16;6:1069455. doi: 10.3389/frai.2023.1069455 (PMC9979214; doi:10.3389/frai.2023.1069455)
Supplement: Supplementary file 1 [file Data_Sheet_1.PDF]

Consent is no
If this question **is exactly**
No
THEN PERFORM THE FOLLOWING ACTION
Show message: You have chosen to not continue with the questionnaire. Thank you for your time.
End survey

Q124

I agree to participate in the study and is informed about how the anonymous information will be used for research:

- Yes
- No

-- PAGE BREAK --

Q9

Importance and Satisfaction

In a general perspective, activities may fulfil different aims, or needs, depending on what is important to us.

The following 15 questions are about some things that you may find important and how happy you are with the extent you are engaging in activities.

Q10

How important is it to you that you have frequent contact with close friends and family?

Not important | Most important

Q94

To what extent are you able to have contact you have with close friends and family?

Too litte extent | Too much extent

Q49

How important is to you to keep up with what happens in society?

Not important | Most important

Q95

To what extent are able to keep up with what happens in society?

Too little extent | Too much extent

Q50

How important is it to you to exercise and/or do other physical activities regularly?

Not important — Most important

Q96

How satisfied are you with the extent you exercise, or are physically active?

Not satisfied — Very satisfied

Q97

How satisfied are you with your emotional wellbeing?

Not satisfied — Very satisfied

Q51

How important is it to you to reduce your worries, anxiety and or/stress?

Not important — Most important

Q52

How important is it to you to be able to perform the activities you want to do?

Not important — Most important

Q98

To what extent are you able to do the things you want to do?

Too little extent — Too much extent

Q100

How satisfied are you with your sleep routines at the moment?

Not satisfied — Very satisfied

Q55

How important is it for you to find time for recovery and increasing energy?

Not important — Most important

Q99

To what extent do you have a good/healthy proportion of activities ongoing at the moment?

Too little extent | Too much extent

Q53

How important is it to you to have fun and being entertained?

Not important | Most important

Q54

How important is it to you to feel safe and secure?

Not important | Most important

-- PAGE BREAK --

Q11

Behaviour Change

To improve health, there are some behaviors, or habits that are targeted by different kinds of health interventions. In this work we are interested in physical activity, stress, alcohol consumption, tobacco use. The following questions relate to these behaviours.

Q117

PHYSICAL ACTIVITY

Q12

How important is it to change your behaviour relating to physical activity? Please, assign the level of importance on a scale of 0-10, where 10 is most important and 0 is not important.

0 | 10

Q33

I want to exercise or do other physical activity because: (you can chose more than one option)

- [ ] I want to improve my health
- [ ] Research shows that physical activity prevents many diseases
- [ ] I want to reduce pain
- [ ] It is relaxing
- [ ] It makes me feel good
- [ ] It gives energy
- [ ] It is a social thing
- [ ] I have to because I sit still all day at work
- [ ] I have always done it, it is a habit
- [ ] Other:

Q34

I don’t exercise/or do physical activity because: (you can chose more than one option)

- [ ] I have never done it regularly, it is not a habit
- [ ] I cannot find the time for it
- [ ] I do not think that it is fun
- [ ] I have too much pain, or other physical condition that stops me
- [ ] The weather is not good
- [ ] It is too expensive to do the things I want to do
- [ ] I would like to do it with others, who are not available
- [ ] Other:

Q35

How prepared are you right now to increase your physical activity? Please, assign the level of preparedness on a scale of 0-10, where 0 is not at all prepared and 10 is completely prepared.

0 10

Q36

Physical activities include both organized activities such as participating in group exercises and sports of different kinds, and hobby activities such as gardening and construction work. What physical activity would you do to improve your health?

Q37

With whom would you typically do the activity? (you can chose more than one option)

- [ ] Partner
- [ ] Child(ren)
- [ ] Pet(s)
- [ ] Extended family
- [ ] Friends(s)
- [ ] Colleague(s)
- [ ] Other
- [ ] None

Q38

What motivates the activity? (you can chose more than one option)

- [ ] It gives energy
- [ ] It's fun, entertaining
- [ ] Rest and recover
- [ ] Others' expectations
- [ ] Obligations
- [ ] Improve physical well-being
- [ ] Nurture relationships with immediate family
- [ ] Nurture relationships with friends and social network
- [ ] Keep up with society
- [ ] Improve emotional well-being
- [ ] Other:

Q39

How often would you do you do the activity?

- ( ) More than once per day
- ( ) Every day
- ( ) A few times per week
- ( ) Once a week
- ( ) Once every other week

Q40

For how long would you do the activity?

- [ ] Minutes:

- [ ] Hours:

Q41

How intensively would you do the activity?

- High
- Moderate
- Low

Q42

Please, define a small, intermediate, easy-to-achieve goal that could help you to increase physical activity:

Q109

If you decide to increase your physical activity, how confident are you in your ability to succeed?

0 10

-- PAGE BREAK --

Q114

STRESS

The following questions relate to managing stress.

Q43

How important is it for you right now to change your behaviour relating to stress? Please, assign the level of importance on a scale of 0-10, where 10 is most important and 0 is not important.

0 10

Q44

Activities that function as recovery activities are important to identify when finding strategies to manage stress. Recovery activities are different between individuals. What activity besides sleep would you do to recover from stress and re-gain energy? (you can chose more than one option)

- [ ] Reading a book
- [ ] Watching movies/TV-series
- [ ] Reading news/watch documentaries
- [ ] Listening to, or playing music
- [ ] Mindfulness exercises such as meditation
- [ ] Spend time in nature
- [ ] Spend time with friends or family
- [ ] Spend time with pets or other animals
- [ ] Spend time on social media
- [ ] Do physical activities such as taking walks or exercise
- [ ] Do physical activities such as gardening or carpeting
- [ ] Do handicrafts
- [ ] Paint, draw
- [ ] Attend cultural events (football, music, exhibitions, etc)
- [ ] Other

Q125

Which one of the selected actvities in previous question works best for you as recovery activity?

Q45

Would you do the activity together with somebody, if so, whom? (you can chose more than one option)

- [ ] Partner
- [ ] Child(ren)
- [ ] Pet(s)
- [ ] Extended family
- [ ] Friends(s)
- [ ] Colleague(s)
- [ ] Other
- [ ] None

Q46

What motivates the activity? (you can chose more than one option)

- It gives energy
- It’s fun, entertaining
- Rest and recover
- Others’ expectations
- Obligations
- Improve physical well-being
- Nurture relationships with immediate family
- Nurture relationships with friends and social network
- Keep up with society
- Improve emotional well-being
- Other:

Q47

How often do you do the activity?

- More than once per day
- Every day
- A few times per week
- Once a week
- Once every other week

Q48

For how long would you do the activity?

- Minutes:
- Hours:

Q110

If you decide to take action to lower your stress, how confident are you in your ability to succeed?

0 10

-- PAGE BREAK --

Q116

ALCOHOL INTAKE

Alcohol

If this question **is exactly**

- Yes

THEN PERFORM THE FOLLOWING ACTION

Show the following questions:

- How important is it for you right now to decrease your alcohol consumption? Please, assign the level of importance on a scale of 0-10, where 10 is most important and 0 is not important.
- I drink alcohol because: (you can chose more than one option)
- I would like to reduce alcohol consumption because: (you can chose more than one option)
- How prepared are you right now to decrease your alcohol consumption? Please, assign the level of preparedness on a scale of 0-10, where 0 is not at all prepared and 10 is completely prepared.
- How would you define a small, in your perspective relatively easy-to-achieve, goal that could help to reduce alcohol consumption? Some examples are shown, please, define your own, which may be more relevant:
- If you decide to decrease your alcohol consumption, how confident are you in your ability to succeed?

Q16

Do you drink alcohol?

- Yes
- No

Alcohol

If Do you drink alcohol? **is exactly**

- Yes

THEN PERFORM THE FOLLOWING ACTION

Show this question

Q31

How important is it for you right now to decrease your alcohol consumption? Please, assign the level of importance on a scale of 0-10, where 10 is most important and 0 is not important.

0 10

Alcohol
If Do you drink alcohol? **is exactly**
- Yes

THEN PERFORM THE FOLLOWING ACTION

Show this question

Q15

I drink alcohol because: (you can chose more than one option)

- [ ] It is a social thing
- [ ] It is a way to relax
- [ ] I need to occupy my hands with something
- [ ] It is a way to reduce pain or other discomfort
- [ ] I have always done it, it is a habit
- [ ] I like the taste
- [ ] Other:

Alcohol
If Do you drink alcohol? **is exactly**
- Yes

THEN PERFORM THE FOLLOWING ACTION

Show this question

Q18

I would like to reduce alcohol consumption because: (you can chose more than one option)

- [ ] Research shows risk for cancer and other health aspects
- [ ] People I care about object
- [ ] It causes discomfort
- [ ] It is expensive
- [ ] Other:

Alcohol
If Do you drink alcohol? **is exactly**
- Yes

THEN PERFORM THE FOLLOWING ACTION

Show this question

Q19

How prepared are you right now to decrease your alcohol consumption? Please, assign the level of preparedness on a scale of 0-10, where 0 is not at all prepared and 10 is completely prepared.

0 10

Alcohol
If Do you drink alcohol? **is exactly**
Yes

THEN PERFORM THE FOLLOWING ACTION

Show this question

Q32

How would you define a small, in your perspective relatively easy-to-achieve, goal that could help to reduce alcohol consumption? Some examples are shown, please, define your own, which may be more relevant:

- [ ] Refrain from having alcohol at home
- [ ] Chose non-alcoholic drinks for meals more often [add number of times per week]
- [ ] Drink fewer glasses when visiting a bar/pub/restaurant: [add number]
- [ ] Chose different venues to meet friends/people instead of a bar/pub [add number of times per week/month]
- [ ] Chose non-drinking friends more often
- [ ] Exchange the habit with something else, which occupy hands [specify what that could be]
- [ ] Other more relevant:

Alcohol
If Do you drink alcohol? **is exactly**
Yes

THEN PERFORM THE FOLLOWING ACTION

Show this question

Q108

If you decide to decrease your alcohol consumption, how confident are you in your ability to succeed?

0 10

-- PAGE BREAK --

Q115

TOBACCO USE

Tobacco

If this question **is exactly**

- Yes

THEN PERFORM THE FOLLOWING ACTION

Show the following questions:

- How important is it for you right now to decrease/quit using tobacco? Please, assign the level of importance on a scale of 0-10, where 10 is most important and 0 is not important.
- I use tobacco because: (you can chose more than one option)
- I would like to refrain from tobacco use because: (you can chose more than one option)
- How prepared are you right now to decrease decrease or quit using tobacco? Please, assign the level of preparedness on a scale of 0-10, where 0 is not at all prepared and 10 is completely prepared.
- How would you define a small goal that could help to reduce tobacco use? Some examples are shown, please, define own that may be more relevant:
- If you decide to decrease or end your tobacco consumption, how confident are you in your ability to succeed?

Q20

Do you use tobacco?

- ( ) Yes
- ( ) No

Tobacco

If Do you use tobacco? **is exactly**

- Yes

THEN PERFORM THE FOLLOWING ACTION

Show this question

Q21

How important is it for you right now to decrease/quit using tobacco? Please, assign the level of importance on a scale of 0-10, where 10 is most important and 0 is not important.

0 — 10

Tobacco

If Do you use tobacco? **is exactly**

- Yes

THEN PERFORM THE FOLLOWING ACTION

Show this question

Q28

I use tobacco because: (you can chose more than one option)

- [ ] It is a social thing
- [ ] It is a way to relax
- [ ] I need to occupy my hands with something
- [ ] It is a way to reduce pain or other discomfort
- [ ] I have always done it, it is a habit
- [ ] I like the taste
- [ ] Other:

Tobacco
If Do you use tobacco? **is exactly**
- Yes

THEN PERFORM THE FOLLOWING ACTION

Show this question

Q29

I would like to refrain from tobacco use because: (you can chose more than one option)

- [ ] Research shows risk for cancer and other health aspects
- [ ] People I care about object
- [ ] It causes discomfort
- [ ] It is expensive
- [ ] Other:

Tobacco
If Do you use tobacco? **is exactly**
- Yes

THEN PERFORM THE FOLLOWING ACTION

Show this question

Q30

How prepared are you right now to decrease decrease or quit using tobacco? Please, assign the level of preparedness on a scale of 0-10, where 0 is not at all prepared and 10 is completely prepared.

0 10

Tobacco
If Do you use tobacco? **is exactly**
- Yes

THEN PERFORM THE FOLLOWING ACTION

Show this question

Q27

How would you define a small goal that could help to reduce tobacco use? Some examples are shown, please, define own that may be more relevant:

- [ ] Refrain from having cigarettes at home
- [ ] Smoke fewer cigarettes when visiting a bar/pub/restaurant: [add number]
- [ ] Exchange the habit with something else, which occupy hands [specify new habit/hobby]
- [ ] Other more relevant:

Tobacco
If Do you use tobacco? **is exactly**
‣ Yes

THEN PERFORM THE FOLLOWING ACTION

Show this question

Q107

If you decide to decrease or end your tobacco consumption, how confident are you in your ability to succeed?

0 10

-- PAGE BREAK --

Q113

A few final questions

Q126

A digital coach application can have different roles when supporting a user to maintain or change behaviours (e.g., when quitting smoking). What role or roles would you like that such digital tool would take on?

- [ ] An assistant that keeps track of your information and reminds you about what you want to be reminded about
- [ ] A coach, similar to a personal trainer who challenges and encourages you to do things
- [ ] A kind of health expert, which informs about current state of knowledge and gives advice
- [ ] A companion, that is more like a friend, keeping you company and is on your side
- [ ] Other:

Q127

Please, motivate your choices and give examples of some situations where the digital tool acts like you want it do do.

Q111

Age
