# Supplementary material for: Argument-based human–AI collaboration for supporting behavior change to improve health
Source: Front Artif Intell. 2023 Feb 16;6:1069455. doi: 10.3389/frai.2023.1069455 (PMC9979214; doi:10.3389/frai.2023.1069455)
Supplement: Supplementary file 2 [file Data_Sheet_2.PDF]

Consent is no

If this question **is exactly**

- No

THEN PERFORM THE FOLLOWING ACTION

Show message: You have chosen to not continue with the questionnaire. Thank you for your time.

End survey

Q1

I agree to participate in the study and am informed about how the anonymous information will be used for research:

- Yes
- No

-- PAGE BREAK --

Q2

Importance and Satisfaction

In a general perspective, activities may fulfill different aims, or needs, depending on what is important to us.

The following 15 questions are about some things that you may find important and how happy you are with the extent you are engaging in activities.

Q3

How important is it to you to have frequent contact with close friends and family?

Not important — Most important

Q4

To what extent are you able to have contact with close friends and family?

Too litte extent — Too much extent

Q7

How important is it to you to exercise and/or do other physical activities regularly?

Not important — Most important

Q8

How satisfied are you with the extent you exercise, or are physically active?

Not satisfied — Very satisfied

Q10

How important is it to you to reduce your worries, anxiety and/or stress?

Not important — Most important

Q9

How satisfied are you with your emotional wellbeing?

Not satisfied — Very satisfied

Q11

How important is it to you to be able to perform the activities you want to do?

Not important — Most important

Q12

To what extent are you able to do the things you want to do?

Too little extent — Too much extent

Q13

How satisfied are you with your sleep routines at the moment?

Not satisfied — Very satisfied

Q14

How important is it for you to find time for recovery and increasing energy?

Not important — Most important

Q15

To what extent do you have a good/healthy proportion of activities ongoing at the moment?

Too little extent — Too much extent

Q16

How important is it to you to have fun and be entertained?

Not important — Most important

-- PAGE BREAK --

Q18

Behaviour Change

To improve health, there are some behaviors, or habits that are targeted by different kinds of health interventions. In this work we are interested in physical activity and stress. The following questions relate to these behaviours.

Q19

PHYSICAL ACTIVITY

Q20

How important is it to change your behaviour relating to physical activity? Please, assign the level of importance on a scale of 0-10, where 10 is most important and 0 is not important.

0 10

Q21

I want to exercise or do other physical activity because: (you can choose more than one option)

- ☐ I want to improve my health
- ☐ Research shows that physical activity prevents many diseases
- ☐ I want to reduce pain
- ☐ It is relaxing
- ☐ It makes me feel good
- ☐ It gives energy
- ☐ It is a social thing
- ☐ I have to because I sit still all day at work
- ☐ I have always done it, it is a habit
- ☐ Other:

Q22

I don't exercise/or do physical activity because: (you can choose more than one option)

- ☐ I have never done it regularly, it is not a habit
- ☐ I cannot find the time for it
- ☐ I do not think that it is fun
- ☐ I have too much pain, or other physical condition that stops me
- ☐ The weather is not good
- ☐ It is too expensive to do the things I want to do
- ☐ I would like to do it with others, who are not available
- ☐ Other:

Q23

How prepared are you right now to increase your physical activity? Please, assign the level of preparedness on a scale of 0-10, where 0 is not at all prepared and 10 is completely prepared.

0 10

Q24

Physical activities include both organized activities such as participating in group exercises and sports of different kinds, and hobby activities such as gardening and construction work. What physical activity would you do to improve your health?

Q25

With whom would you typically do the activity? (you can choose more than one option)

- Partner
- Child(ren)
- Pet(s)
- Extended family
- Friend(s)
- Colleague(s)
- Other
- None

Q26

What motivates the activity? (you can choose more than one option)

- It gives energy
- It’s fun, entertaining
- Rest and recover
- Others’ expectations
- Obligations
- Improve physical well-being
- Nurture relationships with immediate family
- Nurture relationships with friends and social network
- Keep up with society
- Improve emotional well-being
- Other:

Q27

How often would you do you do the activity?

- More than once per day
- Every day
- A few times per week
- Once a week
- Once every other week

Q28

For how long would you do the activity?

☐ Minutes:

☐ Hours:

Q29

How intensively would you do the activity?

- ◯ High
- ◯ Moderate
- ◯ Low

Q30

Please, define a small, intermediate, easy-to-achieve goal that could help you to increase physical activity:

Q31

If you decide to increase your physical activity, how confident are you in your ability to succeed? Please, assign the level of confidence on a scale of 0-10, where 0 is not at all confident and 10 is completely confident.

0 10

Q98

What plans do you have to change your physical activity? Choose the statement that best fits your plans.

- ◯ I have always been physically active, and have no intention to stop being physically active
- ◯ I will probably reduce my physical activity during the coming 3 months
- ◯ I have no plans of becoming more physically active during the coming 3 months
- ◯ I plan to be more physically active during the coming 3 months, but I will probably not make the change within the coming four weeks
- ◯ I plan to become more physically active within the coming four weeks

-- PAGE BREAK --

Q32

STRESS

The following questions relate to managing stress.

Q33

How important is it for you right now to change your behaviour relating to stress? Please, assign the level of importance on a scale of 0-10, where 10 is most important and 0 is not important.

0 10

Q34

Activities that function as recovery activities are important to identify when finding strategies to manage stress. Recovery activities are different between individuals. What activity besides sleep would you do to recover from stress and re-gain energy? (you can choose more than one option)

- [ ] Reading a book
- [ ] Watching movies/TV-series
- [ ] Reading news/watch documentaries
- [ ] Listening to, or playing music
- [ ] Mindfulness exercises such as meditation
- [ ] Spend time in nature
- [ ] Spend time with friends or family
- [ ] Spend time with pets or other animals
- [ ] Spend time on social media
- [ ] Do physical activities such as taking walks or exercise
- [ ] Do physical activities such as gardening or carpenting
- [ ] Do handicrafts
- [ ] Paint, draw
- [ ] Attend cultural events (football, music, exhibitions, etc)
- [ ] Other

Q35

Which one of the selected actvities in previous question works best for you as a recovery activity?

Q36

Would you do the activity together with somebody, if so, whom? (you can choose more than one option)

- [ ] Partner
- [ ] Child(ren)
- [ ] Pet(s)
- [ ] Extended family
- [ ] Friend(s)
- [ ] Colleague(s)
- [ ] Other
- [ ] None

Q37

What motivates the activity? (you can choose more than one option)

- [ ] It gives energy
- [ ] It’s fun, entertaining
- [ ] Rest and recover
- [ ] Others’ expectations
- [ ] Obligations
- [ ] Improve physical well-being
- [ ] Nurture relationships with immediate family
- [ ] Nurture relationships with friends and social network
- [ ] Keep up with society
- [ ] Improve emotional well-being
- [ ] Other:

Q38

How often do you do the activity?

- ( ) More than once per day
- ( ) Every day
- ( ) A few times per week
- ( ) Once a week
- ( ) Once every other week

Q39

For how long would you do the activity?

- [ ] Minutes:
- [ ] Hours:

Q40

If you decide to take action to lower your stress, how confident are you in your ability to succeed? Please, assign the level of confidence on a scale of 0-10, where 0 is not at all confident and 10 is completely confident.

0 10

Q99

Do you plan to make changes that may affect your level of stress? Choose the statement that best fits your plans.

- ◯ I have always regularly been able to balance stress levels, e.g., by doing stress related/lowering recovery exercises.
- ◯ I will probably have things to do that may increase levels of stress coming weeks or months
- ◯ I have no plans to start making changes that may affect levels of stress
- ◯ I will probably start making changes that may affect levels of stress within the next 3 months but I will probably not implement them in the next four weeks
- ◯ I have plans to making changes that may affect levels of stress in the next four weeks

-- PAGE BREAK --

Q57

Questions about the digital assistant's roles and behaviour

Q58

A digital coach application can have different roles when supporting a user to maintain or change behaviours. What role or roles would you like such a digital tool to take on?

- ☐ A kind of health expert, which informs about current state of knowledge and gives advice
- ☐ A companion, that is more like a friend, keeping you company and is on your side
- ☐ A coach, similar to a personal trainer who challenges and encourages you to do things
- ☐ An assistant that keeps track of your information and reminds you about what you want to be reminded about
- ☐ Other:

Q59

Please, motivate your choices and give examples of some situations where the digital tool acts like you want it to.

Q63

How brief would you like the coach to be?

- 4. Very
- 3.
- 2.
- 1. Not particularly

Q65

How facts-based would you like the coach to be?

- 4. Very
- 3.
- 2.
- 1. Not particularly

Q64

How friendly would you like the coach to be?

- 5. Very
- 4.
- 3. Neutral
- 2.
- 1. Not particularly

Q66

How empathic would you like the coach to be?

- 5. Very
- 4.
- 3. Neutral
- 2.
- 1. Not particularly

Q67

How challenging (bossy) would you like the coach to be?

- 4. Very
- 3.
- 2.
- 1. Not particularly

-- PAGE BREAK --

Q68

Scenarios

The following questions relate to the digital agent's behaviour in dialogues with Jim in the first scenarios and with Kim in the next-coming scenarios. The behaviour of the agent differs between the nine short scenarios. Please, evaluate the digital agent's behaviour in the following scenarios.

Q84

Scenario 1a: Jim in the Morning

Jim wants to increase physical activity to improve health, manage a pain condition and reduce some body weight. He also wants to reduce stress. He has as a goal to take 20-minutes walks with his dog four times every day.

The main barriers that he faces are that he finds himself too lazy, having too little time and in addition, he does not think exercise is fun.

In the morning the digital agent reminds Jim about planned activities:

Digital agent	Good morning Jim, the following is your plan for today's activities: take a walk with the dog 4 times, 20 minutes. Yesterday you logged 4 times, on average 10 minutes each.
Jim	Okej.

Q85

1. How brief do you find the digital agent to be?

- 4. Very
- 3.
- 2.
- 1. Not particularly

Q86

2. How facts-based do you find the digital agent to be?

- 4. Very
- 3.
- 2.
- 1. Not particularly

Q87

3. How friendly do you find the digital agent to be?

- 5. Very
- 4.
- 3. Neutral
- 2.
- 1. Not particularly

Q88

4. How empathic do you find the digital agent to be?

- 5. Very
- 4.
- 3. Neutral
- 2.
- 1. Not particularly

Q89

5. How challenging (bossy) do you find the digital agent to be?

- 4. Very
- 3.
- 2.
- 1. Not particularly

Q90

6. What role or roles do you find the digital agent to have in this situation? (more than one option could apply)

- A kind of health expert, which informs about current state of knowledge and gives advice
- A companion, that is more like a friend, keeping you company and is on your side
- A coach, similar to a personal trainer who challenges and encourages you to do things
- An assistant that keeps track of your information and reminds you about what you want to be reminded about
- Other:

-- PAGE BREAK --

Q75

Scenario 1b: Jim at Lunch Time

Jim wants to increase physical activity to improve health, manage a pain condition and reduce some body weight. He also wants to reduce stress. He has as a goal to take 20-minutes walks with his dog four times every day.

The main barriers that he faces are that he finds himself too lazy, having too little time and in addition, he does not think exercise is fun.

At lunchtime the digital agent initiates a conversation with Jim that unfolds in the following way:

Digital agent	Hi Jim, lunch time and time to take a walk with the dog!
Jim	I dont have the time!
Digital agent	You want to exercise or do other physical activity because you want to reduce pain, remember?!
Jim	Work is more important now.
Digital agent	The dog would be happy!
Jim	I'll take a longer walk tonight.
Digital agent	Okej, its a plan, I will remind you!

Q70

7. How brief do you find the digital agent to be?

- 4. Very
- 3.
- 2.
- 1. Not particularly

Q71

8. How facts-based do you find the digital agent to be?

- 4. Very
- 3.
- 2.
- 1. Not particularly

Q72

9. How friendly do you find the digital agent to be?

- 5. Very
- 4.
- 3. Neutral
- 2.
- 1. Not particularly

Q73

10. How empathic do you find the digital agent to be?

- 5. Very
- 4.
- 3. Neutral
- 2.
- 1. Not particularly

Q74

11. How challenging (bossy) do you find the digital agent to be?

- 4. Very
- 3.
- 2.
- 1. Not particularly

Q76

12. What role or roles do you find the digital agent to have in this situation? (more than one option could apply)

- A kind of health expert, which informs about current state of knowledge and gives advice
- A companion, that is more like a friend, keeping you company and is on your side
- A coach, similar to a personal trainer who challenges and encourages you to do things
- An assistant that keeps track of your information and reminds you about what you want to be reminded about
- Other:

-- PAGE BREAK --

Q91

Scenario 1c: Jim the Next Morning

Jim wants to increase physical activity to improve health, manage a pain condition and reduce some body weight. He also wants to reduce stress. He has as a goal to take 20-minutes walks with his dog four times every day.

The main barriers that he faces are that he finds himself too lazy, having too little time and in addition, he does not think exercise is fun.

The next morning the digital agent reminds Jim:

Digital agent	Good morning Jim, a small reminder about your planned physical activities for today! It is important to you to improve your physical health, remember? But your activity seems to have decreased lately. It is understandable if it is difficult to find time in periods, but remember that research has shown that physical activity is actually also good for preventing infections and stress! So taking your walks 4 times at least 20 minutes per day is a good start, try to stick to the plan.
Jim	Yes, but I have too little time today.
Digital agent	That's unfortunate, but time will always be short. It will take you only 20 minutes right now to not only make the dog happy but also improve your health! It's a win-win for everyone, and it is the small steps that matter! So the question is how important is it to you to be physically active?
Jim	Very..
Digital agent	Okej! I'm sure you can do it! Just a little more!

Q92

13. How brief do you find the digital agent to be?

- 4. Very
- 3.
- 2.
- 1. Not particularly

Q93

14. How facts-based do you find the digital agent to be?

- 4. Very
- 3.
- 2.
- 1. Not particularly

Q94

15. How friendly do you find the digital agent to be?

- 5. Very
- 4.
- 3. Neutral
- 2.
- 1. Not particularly

Q95

16. How empathic do you find the digital agent to be?

- 5. Very
- 4.
- 3. Neutral
- 2.
- 1. Not particularly

Q96

17. How challenging (bossy) do you find the digital agent to be?

- 4. Very
- 3.
- 2.
- 1. Not particularly

Q97

18. What role or roles do you find the digital agent to have in this situation? (more than one option could apply)

- A kind of health expert, which informs about current state of knowledge and gives advice
- A companion, that is more like a friend, keeping you company and is on your side
- A coach, similar to a personal trainer who challenges and encourages you to do things
- An assistant that keeps track of your information and reminds you about what you want to be reminded about
- Other:

-- PAGE BREAK --

Q77

Scenario 1d: Jim at Lunch Time Again

Jim wants to increase physical activity to improve health, manage a pain condition and reduce some body weight. He also wants to reduce stress. He has as a goal to take 20-minutes walks with his dog four times every day.

The main barriers that he faces are that he finds himself too lazy, having too little time and in addition, he does not think exercise is fun.

The next day at lunchtime the digital agent initiates a conversation with Jim that unfolds in the following way:

Digital agent	Hi Jim, great that you managed to take a walk this morning! I hope you feel great, as great as the dog did!
Jim	Yes, the dog is happy, so it can wait with another long walk until later. I don't have time now.
Digital agent	That's unfortunate, I hope you don't have too much pain?!
Jim	Some, and it does not get better when just working, but, need to work.
Digital agent	I am sorry to hear that, perhaps you can walk around the table, just to move a bit while working?
Jim	Okej, now that I am at it, I'll just take that walk with the dog then!
Digital agent	That's great, the dog will be very happy!

Q78

19. How brief do you find the digital agent to be?

- 4. Very
- 3.
- 2.
- 1. Not particularly

Q79

20. How facts-based do you find the digital agent to be?

- 4. Very
- 3.
- 2.
- 1. Not particularly

Q80

21. How friendly do you find the digital agent to be?

- 5. Very
- 4.
- 3. Neutral
- 2.
- 1. Not particularly

Q81

22. How empathic do you find the digital agent to be?

- 5. Very
- 4.
- 3. Neutral
- 2.
- 1. Not particularly

Q82

23. How challenging (bossy) do you find the digital agent to be?

- 4. Very
- 3.
- 2.
- 1. Not particularly

Q83

24. What role or roles do you find the digital agent to have in this situation? (more than one option could apply)

- A kind of health expert, which informs about current state of knowledge and gives advice
- A companion, that is more like a friend, keeping you company and is on your side
- A coach, similar to a personal trainer who challenges and encourages you to do things
- An assistant that keeps track of your information and reminds you about what you want to be reminded about
- Other:

-- PAGE BREAK --

Q107

Scenario 1e: Morning Time for Jim Again

Jim wants to increase physical activity to improve health, manage a pain condition and reduce some body weight. He also wants to reduce stress. He has as a goal to take 20-minutes walks with his dog four times every day.

The main barriers that he faces are that he finds himself too lazy, having too little time and in addition, he does not think exercise is fun.

Next morning the digital agent reminds Jim again:

Digital agent	Hi Jim, a small morning reminder about your planned physical activities for today! It is important to you to improve your physical health, remember? But your activity sems to have decreased lately. It is understandable if it is difficult to find time in periods, but remember that research has shown that physical activity is actually also good for preventing infections and stress! So taking your walks 4 times at least 20 minutes per day is a good start, try to stick to the plan.
Jim	Yes, but I have too little time today.
Digital agent	That's unfortunate, but time will always be short. So the question is how important is it to you to be physically active?
Jim	Very..
Digital agent	Okej!

Q108

25. How brief do you find the digital agent to be?

- 4. Very
- 3.
- 2.
- 1. Not particularly

Q109

26. How facts-based do you find the digital agent to be?

- 4. Very
- 3.
- 2.
- 1. Not particularly

Q110

27. How friendly do you find the digital agent to be?

- 5. Very
- 4.
- 3. Neutral
- 2.
- 1. Not particularly

Q111

28. How empathic do you find the digital agent to be?

- 5. Very
- 4.
- 3. Neutral
- 2.
- 1. Not particularly

Q112

29. How challenging (bossy) do you find the digital agent to be?

- 4. Very
- 3.
- 2.
- 1. Not particularly

Q113

30. What role or roles do you find the digital agent to have in this situation? (more than one option could apply)

- A kind of health expert, which informs about current state of knowledge and gives advice
- A companion, that is more like a friend, keeping you company and is on your side
- A coach, similar to a personal trainer who challenges and encourages you to do things
- An assistant that keeps track of your information and reminds you about what you want to be reminded about
- Other:

-- PAGE BREAK --

Q100

Scenario 2a: Kim Activates the Digital Agent at Dinner Time

Kim wants to decrease stress caused by work related responsibilities. The importance to do this he rated very high, at the same time his satisfaction was rated very low.

He also thinks physical activity is important but isn't very prepared or confident in achieving this change.

He has a plan to play games with his partner or friends as a recovery activity to alleviate stress three times per week.

The main barriers he has is finding time, and in particular, mutual free time with his partner or friends, since work takes extra time.

Kim initiates a conversation with the digital agent after coming home from work, and the following conversation takes place:

Digital agent	Hey Kim, What's on your mind?
Kim	I feel overwhelmed with the stress I have at work.
Digital agent	Stress can affect your memory and affect your ability to work in the long run, which you probably already are aware of. How much on a scale from 0 to 10?
Kim	8
Digital agent	This is very high, and as high as you have rated the past three weeks. To have high levels of stress over long period of time is not good, it is important to break this and prioritize time to recover. Is it something on your mind that you should/can do/take care of right now?
Kim	No, it is upcoming things next week I am thinking of.
Digital agent	Then it is important to take the opportunity now to rest and recover! How about playing games with your partner or friends again? It usually helps you to de-stress, you know, and it is part of your plans to do this 3 times per week. So far, you have set this up once a week the past month. It would be really good if you can do this more often as you planned to do.
Kim	That's a good idea. If not tonight, we can set up a game night at the weekend.
Digital agent	How about as soon as possible? Could be a short session. Let me know what they say!
Kim	Okej, I will call them.
Digital agent	Great!

Q101

31. How brief do you find the digital agent to be?

- ◯ 4. Very
- ◯ 3.
- ◯ 2.
- ◯ 1. Not particularly

Q102

32. How facts-based do you find the digital agent to be?

- ○ 4. Very
- ○ 3.
- ○ 2.
- ○ 1. Not particularly

Q103

33. How friendly do you find the digital agent to be?

- ○ 5. Very
- ○ 4.
- ○ 3. Neutral
- ○ 2.
- ○ 1. Not particularly

Q104

34. How empathic do you find the digital agent to be?

- ○ 5. Very
- ○ 4.
- ○ 3. Neutral
- ○ 2.
- ○ 1. Not particularly

Q105

35. How challenging (bossy) do you find the digital agent to be?

- ○ 4. Very
- ○ 3.
- ○ 2.
- ○ 1. Not particularly

Q106

36. What role or roles do you find the digital agent to have in this situation? (more than one option could apply)

- ☐ A kind of health expert, which informs about current state of knowledge and gives advice
- ☐ A companion, that is more like a friend, keeping you company and is on your side
- ☐ A coach, similar to a personal trainer who challenges and encourages you to do things
- ☐ An assistant that keeps track of your information and reminds you about what you want to be reminded about
- ☐ Other: ______

-- PAGE BREAK --

Q114

Scenario 2b: Kim, The Next Morning

Kim wants to decrease stress caused by work related responsibilities. The importance to do this he rated very high, at the same time his satisfaction was rated very low.

He also thinks physical activity is important but isn't very prepared or confident in achieving this change.

He has a plan to play games with his partner or friends as a recovery activity to alleviate stress three times per week.

The main barriers he has is finding time, and in particular, mutual free time with his partner or friends, since work takes extra time.

The next morning after Kim has spoken with his partner and friends he initates a dialogue with the digital agent:

Digital Agent	Hey Kim! How are you feeling now?
Kim	Not so good. My friends aren't free! Still feeling quite stressful…
Digital Agent	I can see how difficult it is for you. But let's not give up just yet, maybe we can alter the number of times you plan to play games with your friends. It'll still be something. What do you say?
Kim	But less than 3 times per week would lead me to still feel stress and would lead me to work even more if I don't have something to occupy myself with.
Digital Agent	Very natural to feel that way, Kim. Let's approach it this way, on the days your friends or partner aren't free for game night, would you be up for doing something else for recovery such as exercise?
Kim	I don't know. Why? Do you think this can help?
Digital Agent	Well, research suggests that regular exercise can improve your mood and lower symptoms of stress and mild depression. Plus, you think physical exercise is important and this could be a great time to start! Exercise can improve your quality of life, not only mentally but also physically, as well!
Kim	Actually, that doesn't sound so bad. So, a choice between game night or exercise when the game night option is out of the question, eh? I don't know…game night still sounds more fun…
Digital Agent	Completely understandable. But remember, we also want to exercise, and this is a proven method to alleviate stress and it'll only take 10 minutes per day.
Kim	That doesn't sound too bad, actually. If it is as you say it is, then exercise can be an option.
Digital Agent	There we go! You've done so much before and I'm sure you can do this!
Kim	Yes, I think I can do it! Why not?
Digital Agent	Yes, why not?! :)

Q115

37. How brief do you find the digital agent to be?

- 4. Very
- 3.
- 2.
- 1. Not particularly

Q116

38. How facts-based do you find the digital agent to be?

- ◯ 4. Very
- ◯ 3.
- ◯ 2.
- ◯ 1. Not particularly

Q117

39. How friendly do you find the digital agent to be?

- ◯ 5. Very
- ◯ 4.
- ◯ 3. Neutral
- ◯ 2.
- ◯ 1. Not particularly

Q118

40. How empathic do you find the digital agent to be?

- ◯ 5. Very
- ◯ 4.
- ◯ 3. Neutral
- ◯ 2.
- ◯ 1. Not particularly

Q119

41. How challenging (bossy) do you find the digital agent to be?

- ◯ 4. Very
- ◯ 3.
- ◯ 2.
- ◯ 1. Not particularly

Q120

42. What role or roles do you find the digital agent to have in this situation? (more than one option could apply)

- ☐ A kind of health expert, which informs about current state of knowledge and gives advice
- ☐ A companion, that is more like a friend, keeping you company and is on your side
- ☐ A coach, similar to a personal trainer who challenges and encourages you to do things
- ☐ An assistant that keeps track of your information and reminds you about what you want to be reminded about
- ☐ Other: ____________

-- PAGE BREAK --

Q121

Scenario 2c: Kim Activates the Digital Agent at Dinner Time, again

Kim wants to decrease stress caused by work related responsibilities. The importance to do this he rated very high, at the same time his satisfaction was rated very low.

He also thinks physical activity is important but isn't very prepared or confident in achieving this change.

He has a plan to play games with his partner or friends as a recovery activity to alleviate stress three times per week.

The main barriers he has is finding time, and in particular, mutual free time with his partner or friends, since work takes extra time.

Kim initiates a conversation with the digital agent after coming home from work, and the following conversation takes place:

Digital Agent	Hey Kim, What's on your mind?
Kim	I feel overwhelmed with the stress I have at work.
Digital Agent	That's understandable. From a scale from 0 to 10, how stressed are you feeling?
Kim	8
Digital Agent	That's very high and compared to your last entries, this is the highest it's been. Your last entry was 6 and the suggested healthy stress level should be much less. Is it something on your mind that can be taken care of now?
Kim	No, it is upcoming things next week I am thinking of.
Digital Agent	That's unfortunate but I'm here for you. May I suggest playing games with your friends again? Last time you played a game with friends, your stress levels decreased by 1. As a reminder, your goal was 3 times per week.
Kim	That's a good idea. If not tonight, we can set up a game night at the weekend.
Digital Agent	A short game session might be possible for your friends. Possibly a call now could be better. I'd be happy to hear what they say.
Kim	Okay! I will call them!
Digital Agent	Great!

Q122

43. How brief do you find the digital agent to be?

- 4. Very
- 3.
- 2.
- 1. Not particularly

Q123

44. How facts-based do you find the digital agent to be?

- 4. Very
- 3.
- 2.
- 1. Not particularly

Q124

45. How friendly do you find the digital agent to be?

- 5. Very
- 4.
- 3. Neutral
- 2.
- 1. Not particularly

Q125

46. How empathic do you find the digital agent to be?

- 5. Very
- 4.
- 3. Neutral
- 2.
- 1. Not particularly

Q126

47. How challenging (bossy) do you find the digital agent to be?

- 4. Very
- 3.
- 2.
- 1. Not particularly

Q127

48. What role or roles do you find the digital agent to have in this situation? (more than one option could apply)

- A kind of health expert, which informs about current state of knowledge and gives advice
- A companion, that is more like a friend, keeping you company and is on your side
- A coach, similar to a personal trainer who challenges and encourages you to do things
- An assistant that keeps track of your information and reminds you about what you want to be reminded about
- Other:

-- PAGE BREAK --

Q128

Scenario 2d: Kim, Again the Next Morning

Kim wants to decrease stress caused by work related responsibilities. The importance to do this he rated very high, at the same time his satisfaction was rated very low.

He also thinks physical activity is important but isn't very prepared or confident in achieving this change.

He has a plan to play games with his partner or friends as a recovery activity to alleviate stress three times per week.

The main barriers he has is finding time, and in particular, mutual free time with his partner or friends, since work takes extra time.

The next morning after Kim has spoken with his partner and friends again the follwing dialogue takes place:

Digital Agent	Hey Kim! How are you feeling now?
Kim	Not so good. My friends aren't free! Still feeling quite stressful…
Digital Agent	Let's not give up just yet, maybe we can alter the number of times you plan to play games with your friends. It's still something.
Kim	But less than 3 times per week would lead me to still feel stress and would lead me to work even more if I don't have something to occupy myself with.
Digital Agent	Understandable. Let's approach it this way, on the days your friends or partner aren't free for game night, let's do some physical activity to fight stress.
Kim	I don't know. Why? Do you think this can help?
Digital Agent	Remember, you think physical exercise is important and is one of your goals. This could be a great time to start! Exercise is not only good for mental health but physical, as well!
Kim	Actually, that doesn't sound so bad. So, a choice between game night or exercise when the game night option is out of the question, eh? I don't know…game night still sounds more fun…
Digital Agent	I get you. But remember, we also want to exercise, and for only 10 minutes per day as a start.
Kim	That doesn't sound too bad, actually. If it is as you say it is, then exercise can be an option.
Digital Agent	All right! I'll remind you tomorrow for sure! You can do it!
Kim	Yes, I think I can do it! Why not?
Digital Agent	Yes, why not?!

Q129

49. How brief do you find the digital agent to be?

- ◯ 4. Very
- ◯ 3.
- ◯ 2.
- ◯ 1. Not particularly

Q130

50. How facts-based do you find the digital agent to be?

- ◯ 4. Very
- ◯ 3.
- ◯ 2.
- ◯ 1. Not particularly

Q131

51. How friendly do you find the digital agent to be?

- ◯ 5. Very
- ◯ 4.
- ◯ 3. Neutral
- ◯ 2.
- ◯ 1. Not particularly

Q132

52. How empathic do you find the digital agent to be?

- ◯ 5. Very
- ◯ 4.
- ◯ 3. Neutral
- ◯ 2.
- ◯ 1. Not particularly

Q133

53. How challenging (bossy) do you find the digital agent to be?

- ◯ 4. Very
- ◯ 3.
- ◯ 2.
- ◯ 1. Not particularly

Q134

54. What role or roles do you find the digital agent to have in this situation? (more than one option could apply)

- ☐ A kind of health expert, which informs about current state of knowledge and gives advice
- ☐ A companion, that is more like a friend, keeping you company and is on your side
- ☐ A coach, similar to a personal trainer who challenges and encourages you to do things
- ☐ An assistant that keeps track of your information and reminds you about what you want to be reminded about
- ☐ Other:

-- PAGE BREAK --

Q69

General questions

Q135

55. What is the highest degree or level of school you have completed?

- Basic education, up to 9 years
- Basic education, completed 9th grade education
- Gymnasium or alike, shorter than 3 years
- Completed education program at gymnasium level, 3 years or longer
- Vocational school: completed education
- University or alike: 1-3 year education without a degree
- University or alike: 3 years or more education with a degree (e.g., Bachelor's, nursing school)
- University or alike: 4-8 year education with or without a degree (e.g., physician, engineer, Master's degree)
- University or alike: completed research education

Q60

Age

Q137

56. What is your country of residence?

Q136

57. What is your nationality?

Q61

Gender

- Female
- Male
- Other
